# Performance Evolution and Microstructure Optimization of Recycled Fine Aggregate Rapid-Hardening Sulfoaluminate Cement Mortar by Nano-SiO_2_ Surface Modification

**DOI:** 10.3390/nano16171051

**Published:** 2026-08-23

**Authors:** Meinan Wang, Shuo Liu, Cong Zhang, Yaning Wu, Liang Wang, Tieming Guo

**Affiliations:** 1 College of Civil Engineering & Architecture, Qingdao Agricultural University, Qingdao 266109, China; 2 Qingdao Branch of Tongyuan Design Group Co., Ltd., Qingdao 266001, China

**Keywords:** nano-modification, recycled fine aggregate, rapid hardening sulfoaluminate cement, microstructure, microhardness

## Abstract

In this study, rapid-hardening sulfoaluminate cement (SAC) was used as cementitious material, and recycled fine aggregates (RFAs) were surface pretreated by immersion in nano-SiO_2_ (NS) suspensions. NS-modified SAC recycled fine aggregate mortars were prepared at three cement–sand ratios (1:1, 1:2 and 1:3) to systematically investigate the regulatory effects of NS concentrations (0%, 1%, 2% and 3%) on macroscopic performance, hydration products and interfacial microstructure. Multi-scale characterizations, including XRD, TG-DTG, SEM-EDS and microhardness tests, were carried out. The testing results show that appropriate NS can optimize SAC hydration by heterogeneous nucleation and the pozzolanic reaction. At a cement–sand ratio of 1:1, the compressive and flexural strengths gradually increase as the NS concentration rises from 0% to 2%. Compared with the control group, the 28 d compressive and flexural strength are enhanced by 19.5% and 16.6%, respectively, the drying shrinkage decreases by 8.0%, and carbonation resistance is obviously improved. Meanwhile, the formation of AFt is promoted, amorphous C-S-H gel accumulates continuously, and the content of Ca(OH)_2_ is gradually consumed by the pozzolanic reaction of NS. For specimens modified with 2% NS, the maximum microhardness reaches 1326 HV, which greatly benefits the mechanical properties of mortar. However, further increasing the NS concentration to 3% triggers nanoparticle agglomeration and reduces effective reactive silica, leading to a decline in hydration products, deteriorated interfacial compactness and reduced mechanical performance. Therefore, 2% can be determined as the optimal NS concentration which can provide a theoretical basis for high-value resource recycling of recycled fine aggregates in SAC mortar.

## 1. Introduction

With the acceleration of global urbanization and the continuous advancement of infrastructure, large amounts of waste concrete are generated during building demolition, road renovation and concrete component renewal. If such construction solid waste is disposed of by open-air stockpiling or land filling over extended periods, it not only occupies valuable land resources but also causes dust pollution and resource wastage, thereby further increasing the ecological and environmental burden [1]. Meanwhile, the large-scale production of cement mortar and concrete continues to increase the demand for natural aggregates. The excessive mining of natural river sand can easily lead to a series of problems, such as river ecological damage, resource shortages, and rising material costs [2,3]. Although manufactured sand can alleviate the shortage of natural river sand to some extent, its production still consumes large amounts of rock resources and energy and may also cause issues such as dust emissions and fluctuations in particle gradation control. Therefore, processing waste concrete through crushing, impurity removal, sieving and grading [4] to use them to partially or fully replace natural aggregates in cement-based materials [5,6] has become an important approach for the resource recovery of construction solid waste. This approach not only effectively reduces the consumption of natural fine aggregate resources but also alleviates the stockpiling pressure of construction waste, promoting the building material industry toward low-carbon, green, and sustainable development [7,8,9].

Recycled fine aggregates are typically produced by crushing waste concrete or mortar [10]. However, due to the adhered old cement paste on their surfaces [11] and the presence of pores, microcracks, and interfacial defects [12,13], their physical properties are generally inferior to those of natural river sand. Compared with natural fine aggregates, recycled fine aggregates generally exhibit higher water absorption, lower apparent density, weaker particle strength, and poorer gradation stability, which can adversely affect the workability, mechanical performance, and durability of cement mortar [14]. In addition, the residual old mortar on their surfaces tends to form a weak and porous interfacial transition zone with the new cement paste, thereby weakening the mechanical properties and durability of mortar [15]. Therefore, improving the surface structure of recycled fine aggregates, reducing their porosity and water absorption, and enhancing their bonding with the new cement matrix are critical for improving the overall performance of recycled fine aggregate mortar [16].

To address the performance deterioration of recycled aggregates [17], various modification methods have been proposed, including mechanical grinding [18], acid washing, carbonation treatment [19,20] and chemical impregnation [21]. Xia et al. [22] recovered natural aggregates and spalling mortar from crushed concrete through freeze–thaw separation and sodium silicate mineralization and found that this method could remove adhered mortar and fill pores and cracks by forming silica gel and C-S-H gel, thus improving the mechanical properties of recycled mortar. Chen et al. [23] further revealed that carbonation treatment could optimize the pore structure of recycled fine aggregates and enhance interfacial compactness, thereby reducing chloride ion diffusion in UHPC and improving durability. These studies demonstrate that targeted modification of surface pores and interfacial defects is an effective strategy for improving the application performance of recycled fine aggregates.

Among various modification techniques, nanomaterial modification has attracted extensive attention, owing to its high reactivity, strong filling capacity, and effective regulation of interfacial properties [24,25]. Related studies indicate that nanomaterials mainly play a role in building material modification through pore filling, interface strengthening, nucleation induction, and microstructure optimization [26,27,28]. They can improve building material performance from multiple scales, such as pore structure regulation [29], interface performance enhancement [30], and microstructure optimization [31], thereby providing new research ideas for surface reinforcement of recycled fine aggregates. In particular, nano-SiO_2_, with its small particle size, large specific surface area, and high pozzolanic activity, can improve the microstructure of cement-based materials through physical filling, nucleation, and pozzolanic reactions [32]. Shi et al. [33] and Ding et al. [34] found that nano-SiO_2_ promoted C-(A)-S-H gel formation, refined pore structures, and improved the early-age and long-term mechanical performance of solid-waste-based cementitious systems. Wang et al. [35] further found that nano-SiO_2_ surface modification enhanced the rubber–cement interface and improved the strength of rubberized concrete, while Yi et al. [36] showed that carbonation combined with hydrophobic nano-SiO_2_ reduced the water absorption and porosity of recycled aggregates and optimized the interfacial transition zone of recycled aggregate concrete. Therefore, nano-SiO_2_ pretreatment is expected to improve the surface pore structure and interfacial bonding of recycled fine aggregates, thereby enhancing the workability, mechanical properties, and microstructural compactness of recycled fine aggregate mortar.

However, the modification effect of nano-SiO_2_ on recycled fine aggregates is closely related to its solution concentration rather than simply increasing with dosage [37]. At an appropriate concentration, nano-SiO_2_ can uniformly attach to aggregate surfaces, fill pores and microcracks, and react with Ca(OH)_2_ to form additional C-S-H gels, thereby improving the interfacial transition zone [38]. In contrast, excessive nano-SiO_2_ may agglomerate, form uneven coatings, increase water demand, and reduce mortar workability and strength development [39]. Therefore, clarifying the effect of nano-SiO_2_ solution concentration is essential for optimizing the modification of recycled fine aggregates and improving recycled fine aggregate mortar performance.

In this study, recycled fine aggregates were modified by immersion in nano-SiO_2_ solutions with different concentrations and subsequently used to prepare rapid-hardening sulfoaluminate cement mortar specimens. The effects of nano-SiO_2_ solution concentration on strength development, shrinkage and carbonation performance of SAC-based recycled fine aggregate mortar were systematically evaluated through macro tests. Meanwhile, XRD, TG/DTG, SEM-EDS, and microhardness analyses were conducted to characterize the phase composition, micro-morphology, elemental distribution, and interfacial transition zone characteristics of the mortar. Based on the correlation between macroscopic performance and microstructural evolution, the modification mechanism of nano-SiO_2_ on SAC-based recycled mortar was further clarified, providing a theoretical reference for the efficient utilization of recycled fine aggregates in SAC cement mortar.

## 2. Experimental Program

### 2.1. Experimental Raw Materials

#### 2.1.1. Cementitious Material

In this study, rapid hardening sulfoaluminate cement (SAC) is used as cementitious material, provided by Tangshan Polar Bear Building Materials Co., Ltd. (Tangshan city, Hebei, China), to compensate for the early strength of recycled fine aggregate mortar. The physical performance parameters and the XRF chemical composition of SAC are shown in Table 1 and Table 2, respectively.

#### 2.1.2. Recycled Fine Aggregates

Compared with natural fine aggregates, recycled fine aggregates (RFAs) exhibit a loose internal structure with abundant inherent micropores and microcracks. The residual old mortar adhered to the particle surface further increases its porosity, water absorption, and crushing index. In this study, class-II RFAs were adopted as fine aggregates. These class-II RFAs were produced by crushing ordinary waste concrete, with a maximum particle size of 4.75 mm and continuous particle-size distribution. Their main mineral components are quartz, calcite and residual cement hydrates. The tested bulk density of 1457 kg/m^3^, apparent density of 2538 kg/m^3^, fineness modulus of 2.52, 24 h water absorption of 7.05%, and crushing index of 16.14% meet the standard requirements of GB/T 25176-2010 “Recycled fine aggregate for concrete and mortar” [40]. Owing to its porous microstructure, RFAs present obvious water absorption and desorption characteristics, which inevitably increase the mixing water consumption of mortar. Moreover, inferior interfacial transition zones are easily formed between aggregates and cement paste, thereby deteriorating the compactness and mechanical properties of hardened mortar. Therefore, surface impregnation modification using nano-SiO_2_ suspension is adopted to fill surface pores and mitigate interfacial defects, which serves as an effective approach to improve the macroscopic performance and micro-structure of recycled aggregate mortar.

#### 2.1.3. Nano-SiO_2_

Nano-silica (NS) possesses abundant unsaturated chemical bonds and silanol groups (Si-OH) on its surface. These sufficient active sites can not only accelerate the hydration reaction of cement but also effectively improve the bonding performance of the interfacial transition zone (ITZ) in recycled mortar. The NS used in this experiment was supplied by Nanjing Baokete New Materials Co., Ltd., and is a white amorphous powder with a spherical particle structure. It has a particle size of 10–30 nm, a specific surface area of 237 m^2^/g, an apparent density of 2.58 g/cm^3^ and purity higher than 99.9%.

**Figure 1:**
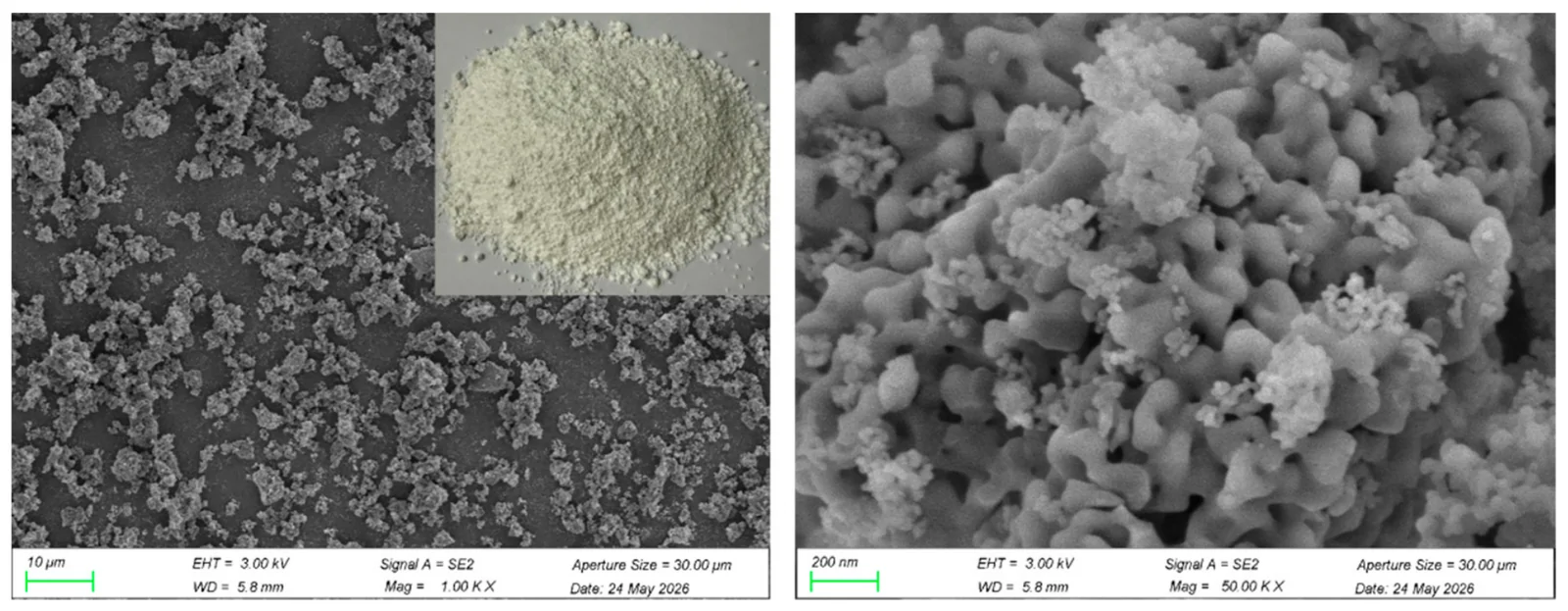
Appearance and SEM micro-morphology of NS.

Because NS powder is prone to agglomeration and flocculation, the ultrasonic dispersion method was adopted to prepare NS suspension. A certain amount of NS powder was mixed evenly with deionized water in advance and then dispersed for 20 min at a constant water bath temperature of 25 °C using a 500 W ultrasonic disperser under the intermittent mode of 3 s on and 2 s off. After dispersion, the suspension was left to stand for 3 min to remove air bubbles. When the sediment volume at the bottom was less than 5% of the total volume, NS suspensions with different mass concentrations (1%, 2%, and 3%) were eventually prepared, as shown in Figure 1. The 1%, 2% and 3% labels refer to the mass fraction of nano-SiO_2_ powder relative to deionized water for suspension preparation. In the modified RFA experiment, all NS in the mortar was pre-loaded onto the RFA surface by soaking modification, and no additional NS powder was mixed into the cement paste during mortar casting. The NS suspension concentration only represents the preparation parameter of modification liquid.

To quantify the actual NS adsorbed by RFAs after soaking and filtration, parallel adsorption tests were carried out. A total of 1000 g of saturated-surface-dry (SSD) unmodified RFAs were immersed in 1000 g of NS suspension at each concentration for 24 h. After filtration of excess suspension, modified RFAs were dried to the SSD condition at 60 °C to record mass increment, and the retained dry NS mass and retention rate were calculated. The NS retention rate refers to the ratio of retained NS mass to total NS added in suspension. After testing and calculation, for each 1000 g of raw RFAs, the total adsorbed solid mass after modification was 12.4 g, 23.7 g and 31.2 g under 1%, 2% and 3% NS suspension, while the retained dry NS mass was 8.1 g, 15.6 g and 20.8 g, corresponding to retention rates of 81.0%, 78.0% and 69.3%. The retention rate declines with rising suspension concentration mainly due to nanoparticle agglomeration, as larger NS agglomerates cannot enter internal pores of RFAs and are washed away during filtration, leading to lower adsorption efficiency at 3%.

#### 2.1.4. NS-Modified Recycled Fine Aggregates

To directly quantify the surface modification effect of nano-SiO_2_, the physical property indicators of unmodified raw RFAs and different NS-modified RFAs were tested, and the comparative results are listed in Table 3. It can be observed that after NS modification, the nanoparticles fill surface microcracks and capillary pores of RFAs, which significantly optimizes aggregate performance. The 2% NS suspension yields the best improvement. The water absorption decreases from 7.05% to 5.53%, apparent density increases from 2538 kg/m^3^ to 2588 kg/m^3^, bulk density increases from 1457 kg/m^3^ to 1523 kg/m^3^, and crushing index falls by 17.3%. When the suspension concentration rises to 3%, massive NS agglomerates cannot fully penetrate into tiny intra-aggregate pores and are partially lost during filtration, resulting in weakened filling efficiency and slightly worse aggregate properties compared with the 2% group.

#### 2.1.5. Polycarboxylate Superplasticizer

The efficient polycarboxylate superplasticizer (PCE) used in this study was prepared by the Shandong Shanshui Cement Group, and the amount of PCE accounts for 1% of the cement amount, with a water reduction rate of 28–30%.

### 2.2. Mix Proportion of NRFA Mortar

In this study, to investigate the mechanical properties, durability, and microstructural evolution of nano-modified recycled fine aggregate mortar (NRFM), four mass concentrations of NS suspension (0%, 1%, 2%, and 3%) and three cement-to-RFA ratios (1:1, 1:2, and 1:3) were designed. All mortar mixtures were adjusted to maintain a target fluidity range of 180–200 mm by regulating the mixing water content. The initial designed water–cement ratio was 0.5. Considering the water absorption characteristics of nano-SiO_2_ and recycled fine aggregates, the actual water content was adjusted appropriately for each group to guarantee identical workability, so as to ensure the comparability of subsequent mechanical property and durability test results. The flexural strength and compressive strength of NRFM were tested at curing ages of 3 d, 7 d, and 28 d, together with a series of durability evaluations. Furthermore, microstructural characterizations, including XRD analysis, TG-DTG, microhardness, and SEM-EDS tests, were conducted to reveal the intrinsic modification mechanism of NRFM. The detailed mix proportions are listed in Table 4. NRFM1, NRFM2 and NRFM3 in Table 4 correspond to mortar groups with cement–NRFA ratios of 1:1, 1:2 and 1:3, respectively. The numbers following the codes denote the concentration of NS suspension. For example, the number in NRFM2-1 represents the mortar mix proportion ratio when the cement/RFA ratio is 1:2 and the concentration of NS suspension is 1.0%. According to the designed mix proportion scheme, the NS-soaked modified recycled fine aggregate mortar (NRFM) can be prepared finally.

### 2.3. Experimental Methods

In this study, the mechanical performance tests of NRFM mortar were conducted in accordance with the GB/T 50081-2019 Standard for Test Methods of Physical and Mechanical Properties of Concrete [41]. The specimens for compressive and flexural strength tests were prepared using standard mortar moulds with a dimension of 40 mm × 40 mm × 160 mm. For every single mix and curing age, 9 specimens were prepared in total (3 independent batches, with 3 parallel specimens per batch). Among them, 3 pieces for flexural test, the 6 broken fragments after flexural failure were divided into two groups for compressive tests. After casting, the specimens were sealed with a film and kept at room temperature for 24 h before demolding. Subsequently, they were cured in a standard curing chamber at a temperature of (20 ± 2) °C and relative humidity of no less than 95%. Upon reaching the specified curing age, the universal testing machine was adopted to test the compressive strength and flexural strength. The flexural test loading rate was set to 0.05 mm/min, the compressive test loading rate was 0.5 MPa/s, and continuous uniform loading was performed until complete failure of the specimen. Standard shrinkage specimens sized 25 mm × 25 mm × 280 mm were used for drying shrinkage tests. For each mix group, 6 specimens (2 batches, with 3 parallel specimens per batch) were prepared. After standard curing for 3 d, specimens were transferred to a drying chamber with a constant temperature and humidity for shrinkage measurement: drying temperature was set to (20 ± 2) °C, and relative humidity was controlled at (60 ± 3)%. A vertical mortar length comparator was used to measure length variation, and shrinkage rates were recorded at 3 d, 7 d, 14 d, 28 d and 91 d. Prismatic specimens of 100 mm × 100 mm × 400 mm were adopted for carbonation tests. Each proportion was prepared with 6 parallel specimens from 2 independent batches. After 28 days of curing, the dried specimens were placed into a concrete carbonation test chamber, where the carbon dioxide concentration was maintained at 18~22%, relative humidity was controlled within 65~75%, and the temperature was kept constant at (20 ± 2) °C. Specimens were taken out after carbonation for 3 d, 7 d, 14 d, 28 d, and a phenolphthalein indicator was used to determine the carbonation depth.

The freeze–thaw test followed the GB/T 50082-2024 Standard for Test Methods of Long-term Performance and Durability of Ordinary Concrete [42]. The specimen dimensions were 100 mm × 100 mm × 400 mm. For each mix proportion, 6 test specimens from 3 independent batches were prepared. For the temperature range of a full cycle, the freezing temperature was −18 ± 2 °C, and the thawing temperature was 5 ± 2 °C. The single-cycle total duration was 4 h, including a 2.5 h freezing stage and a 1.5 h thawing stage. Tap water was used as the circulating medium. Before freeze–thaw circulation, all specimens after 28 d of standard curing were immersed in water at 20 °C for 4 d to reach a saturated-surface-dry state. The initial relative dynamic elastic modulus of each specimen was recorded as the benchmark value (100%). The relative dynamic elastic modulus of all specimens was tested every 25 freeze–thaw cycles. During circulation, surface scaling damage of specimens was observed synchronously, and the test was terminated when the average relative dynamic elastic modulus of one group dropped below 60%.

**Figure 2:**
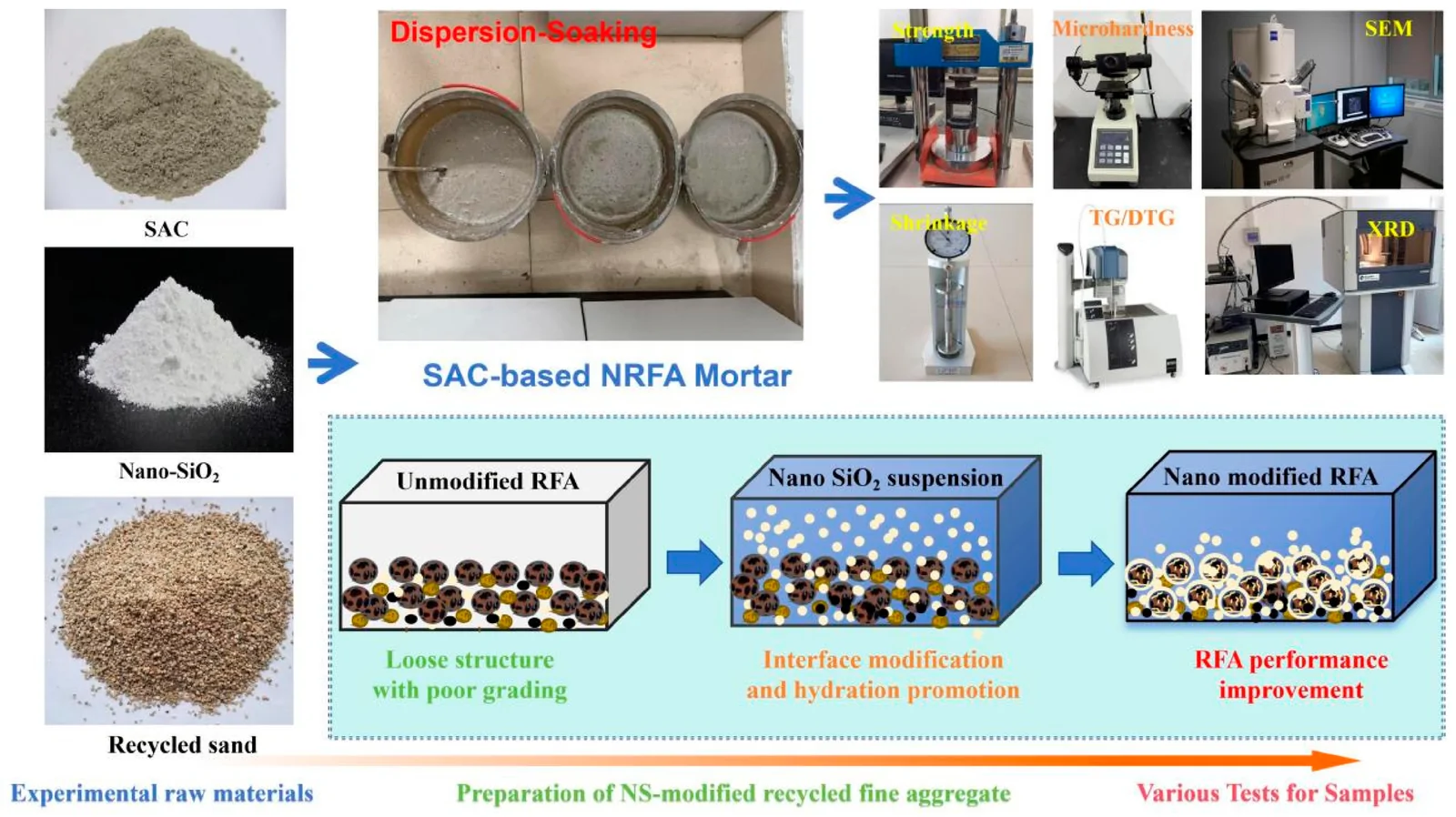
An experimental flowchart and performance testing of NRFM mortar.

For microscopic tests, mortar specimens at the target curing age were cut by a cutting machine to prepare microscopic samples. The cut samples were immersed in acetone solution to terminate hydration. After immersion, the samples were taken out and dried, followed by grinding and polishing treatment. The well-treated mortar samples were ground into powder and sieved through a 200-mesh standard sieve. XRD tests were performed on a Bruker D8 Advance X-ray polycrystalline diffractometer using Cu Kα radiation (λ = 0.15406 nm), operated at 40 kV and 40 mA. Samples were scanned over 2θ = 5–60° with a step size of 0.02° and a scanning rate of 2 °/min, and each test used 2 g of the powder sample. Thermogravimetric and derivative thermogravimetric (TG/DTG) tests were carried out under high-purity nitrogen atmosphere (50 mL/min) using alumina ceramic crucibles. Approximately 10–12 mg of the ground powder sample was heated from 25 °C to 1000 °C at a constant heating rate of 10 °C/min. A TESCAN MIRA LMS-type scanning electron microscope (SEM, manufactured in Czech) was adopted to observe the microscopic morphology of the NRFM mortar, the interfacial transition zone between the recycled fine aggregates and paste, as well as pore distribution. A microhardness tester was used to conduct microhardness tests on polished mortar samples for quantitative analysis on the compactness of internal structure and mechanical uniformity of mortar. A detailed flowchart for the sample preparation and testing equipment is shown in Figure 2.

## 3. Results and Discussion

### 3.1. Compressive Strength of NRFM

**Figure 3:**
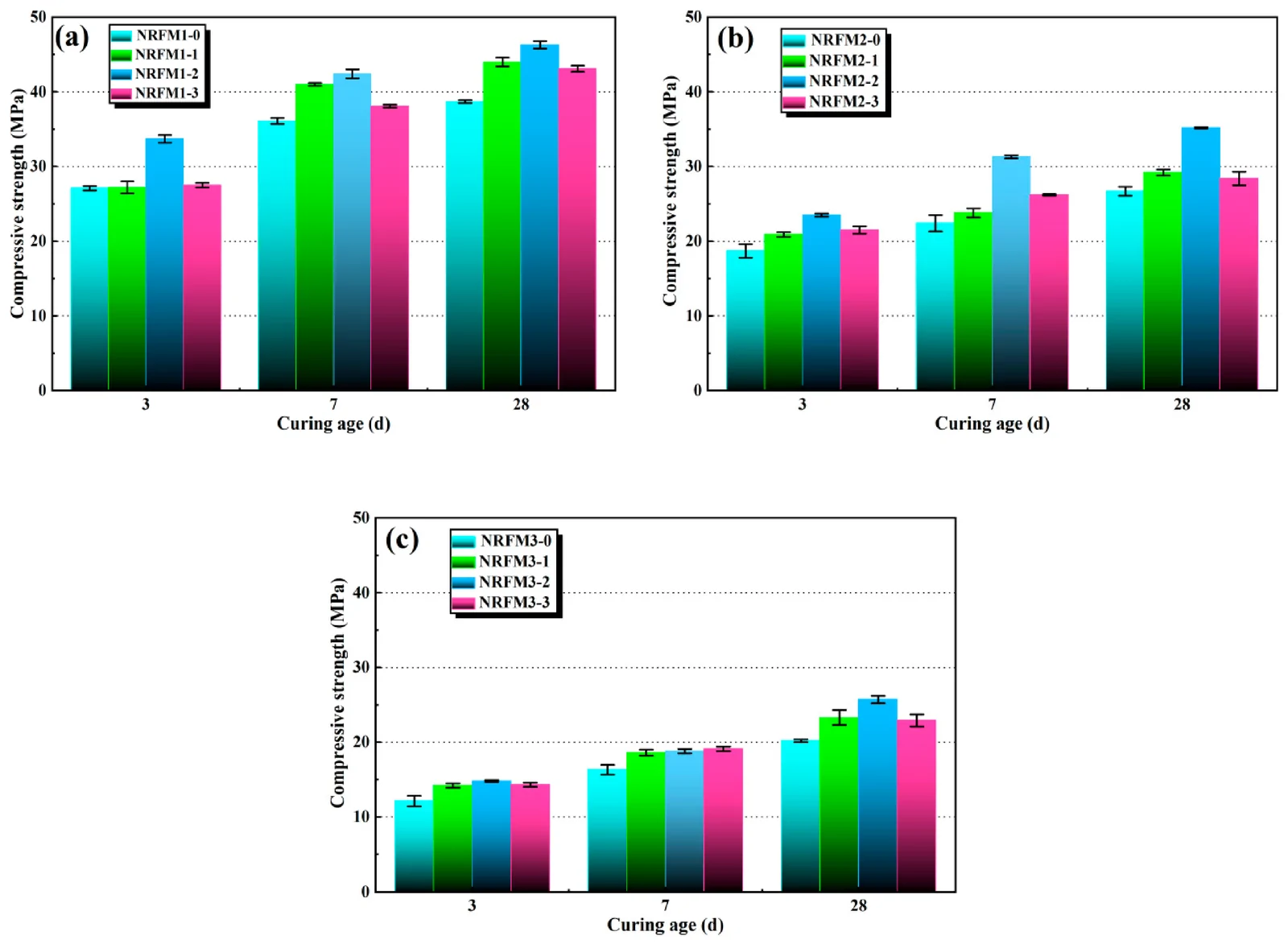
Compressive strengths of different series of NRFM at different cement/sand ratios: (**a**) 1:1; (**b**) 1:2; (**c**) 1:3.

Figure 3a–c shows the compressive-strength development of NRFM at different curing ages and under different cement–sand ratios of 1:1, 1:2 and 1:3. It can be seen that the compressive strength of unmodified recycled fine aggregate mortar declines markedly with a reduction in cement–sand ratio. The compressive strength of NRFM1-0 at 3 d, 7 d and 28 d reaches 27 MPa, 36 MPa and 39 MPa respectively, which is considerably higher than that of NRFM2-0 (19 MPa, 22 MPa, and 27 MPa) and NRFM3-0 (12 MPa, 16 MPa, and 20 MPa). This phenomenon is closely associated with the reduced compactness and deteriorated interfacial transition zone caused by the decreased volume of cement paste. NS effectively enhances the compressive strength of all NRFM groups in a concentration-dependent manner. The strength continuously increases when the NS suspension concentration rises from 0% to 2%, while a strength reduction occurs at 3%, demonstrating that 2% is the optimal content for all tested cement–sand ratios. The 28-day compressive strength of NRFM1-2, NRFM2-2 and NRFM3-2 is 46 MPa, 35 MPa and 26 MPa, increasing by approximately 18%, 29% and 30% compared with the corresponding control groups (0% NS). The strength improvement is attributed to the pozzolanic reaction and microaggregate filling effect of nano-SiO_2_, as nano-scale particles fill micro-cavities on RFA surfaces, reduce the inter-particle void volume, significantly raise the overall packing density of the solid skeleton, and consume Ca(OH)_2_ by the pozzolanic reaction, which densifies the ITZ and refines pore structure. The strength degradation at 3% NS suspension concentration results from the formation of micron-scale agglomerates driven by high surface energy. Such agglomerates cannot exert a nanoscale filling effect, hinder water migration and disrupt uniform formation of hydration products, thereby lowering the overall compactness and mechanical properties of NRFM mortar.

Furthermore, NS presents a more prominent strength compensation effect on mortar with a low cement–sand ratio, and the strength growth rates of NRFM2 and NRFM3 are higher than that of NRFM1. All specimens exhibit the typical rapid early-strength-development characteristic of rapid hardening SAC, and the 3-day strength accounts for 65–75% of the 28-day strength. It indicates that NS modification can ameliorate the mechanical performance of recycled fine aggregate mortar by optimizing cement paste and interfacial microstructure without altering the fundamental hydration process of cement and has superior application prospect in mixtures with low cement–sand ratios of 1:2 and 1:3.

### 3.2. Flexural Strength of NRFM

**Figure 4:**
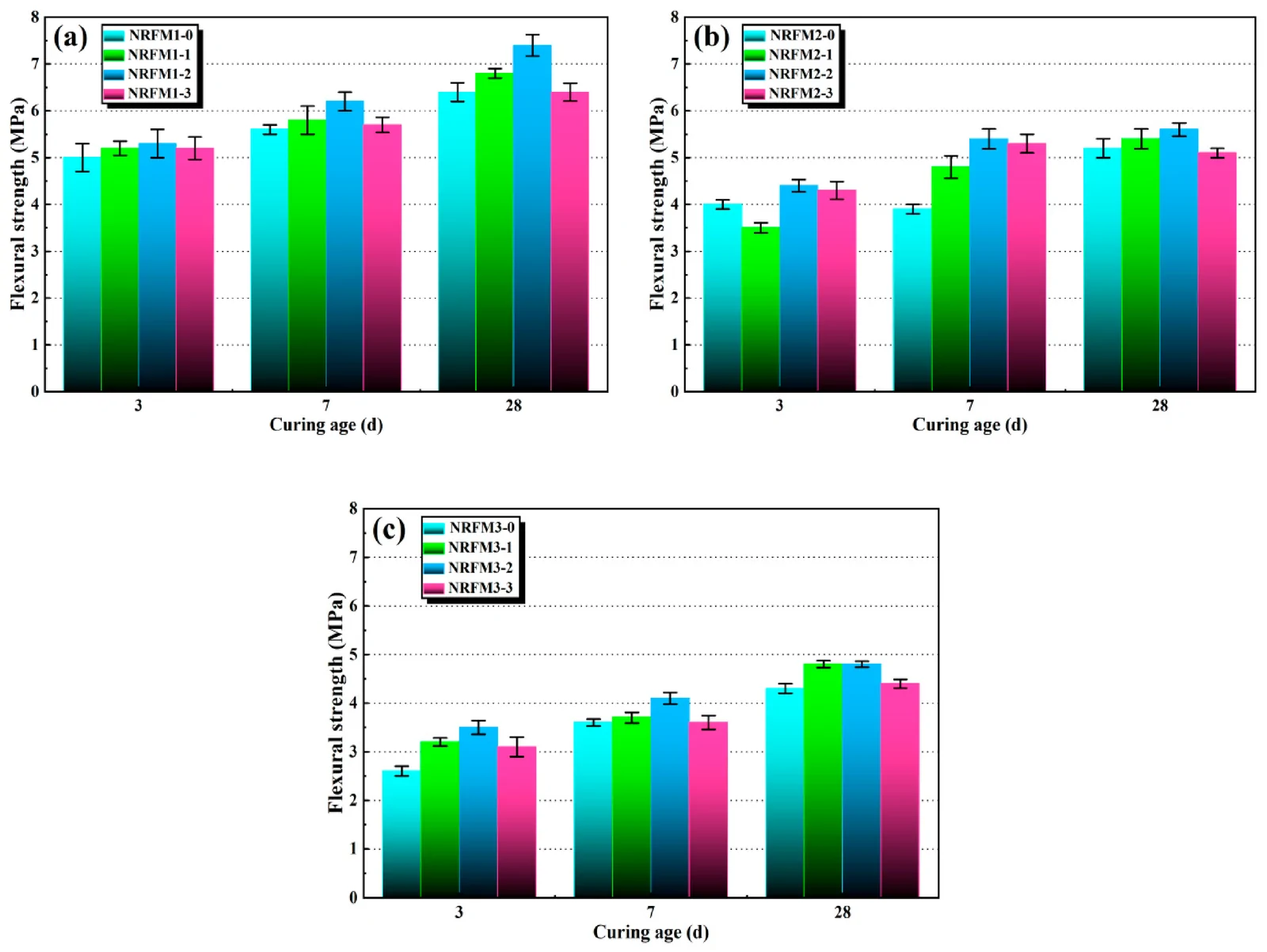
Flexural strengths of different series of NRFM at different cement/sand ratios: (**a**) 1:1; (**b**) 1:2; (**c**) 1:3.

Figure 4 illustrates the flexural strength development of NRFM at different curing ages and under different cement-to-sand ratio conditions. For the unmodified control groups, the reduction in flexural strength with a decreasing cement-to-sand ratio is significantly greater than that of compressive strength. Specifically, the 28 d flexural strength of NRFM3-0 is only 67% of that of NRFM1-0, while the corresponding reduction in compressive strength is 49%. The flexural strength continuously increases as the NS concentration rises from 0% to 2%, followed by a decrease at 3%, indicating that 2% is the optimal content for all cement-to-sand ratios, which is consistent with the trend observed for compressive strength. The 28 d flexural strengths of NRFM1-2, NRFM2-2, and NRFM3-2 reach 7.4 MPa, 5.6 MPa, and 4.8 MPa, representing enhancements of approximately 16%, 8%, and 12% compared with control groups (0% NS). Such improvements are attributed to the pozzolanic reaction and micro-filling effect of NS, thereby improving the flexural performance of the mortar. Notably, when the NS concentration is increased to 3%, the 28 d flexural strength of NRFM1-3 decreases by approximately 14% compared with NRFM1-2, which is far greater than the 6.5% reduction observed in compressive strength. This phenomenon indicates the detrimental effect of excessive NS agglomeration on interfacial bonding, as NS agglomerates act as new stress concentration sites within the ITZ, weakening the mechanical interlocking between aggregates and paste and ultimately leading to the degradation of flexural performance.

### 3.3. Shrinkage Performance of NRFM

**Figure 5:**
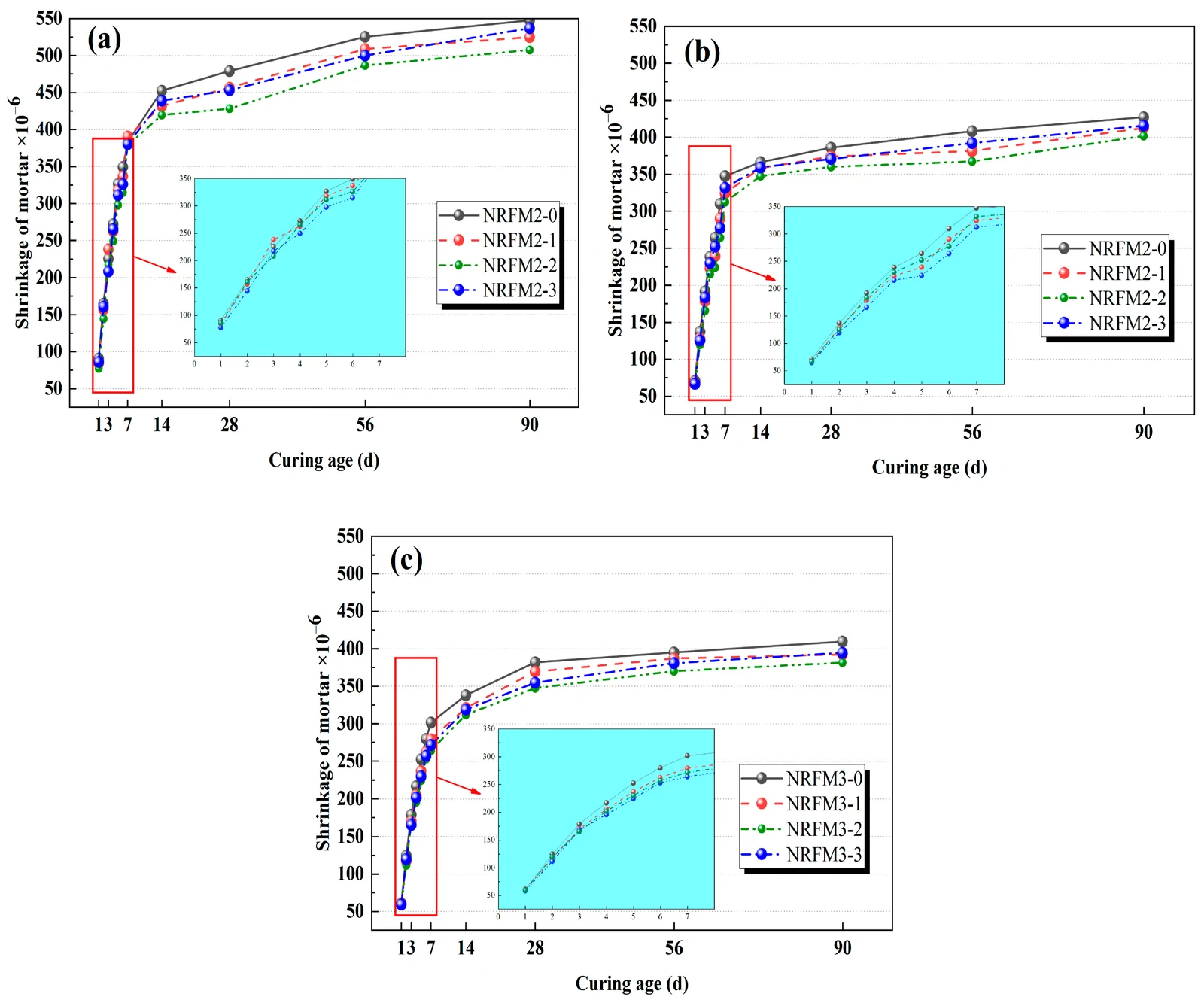
Shrinkage of different series of NRFM at different cement/sand ratios: (**a**) 1:1; (**b**) 1:2; (**c**) 1:3.

Figure 5 shows the shrinkage evolution of NRFMs at different curing ages and under different cement-to-sand ratios. All specimens exhibit a typical characteristic of rapid early-shrinkage development, with more than 70% of the total 90 d shrinkage occurring within the first 14 days, followed by a gradual leveling off. This behavior is directly related to the hydration process and early water-evaporation characteristics of fast-hardening SAC. The porous and water-absorbing nature of RFAs tends to increase the shrinkage of the mortar, while NS modification can significantly reduce the shrinkage in a concentration-dependent manner. As the NS concentration increases from 0% to 2%, the shrinkage value decreases continuously, with a slight rebound observed at 3%, indicating that 2% is the optimal content. Taking the NRFM2 series as an example, the 90 d shrinkage of NRFM2-2 is reduced by approximately 10% compared with the control group NRFM2-0, and NRFM3-2 in the NRFM3 series shows an 8% reduction relative to NRFM3-0. This improvement stems from the micro-aggregate filling effect of NS, which refines the capillary pore structure, fills aggregate pores, and reduces water migration pathways. Meanwhile, additional hydration products formed by the pozzolanic reaction optimize matrix compactness, effectively inhibiting early volume shrinkage of the mortar, thus markedly improving the volume stability of the NRFM. Furthermore, the cement-to-sand ratio significantly affects the shrinkage level, with the NRFM3 series exhibiting overall lower shrinkage values than the NRFM2 series, which is attributed to the reduced amount of hydration products and the consequent lower contribution of paste shrinkage due to the lower content of SAC cementitious materials.

### 3.4. Carbonization Performance of NRFM

**Figure 6:**
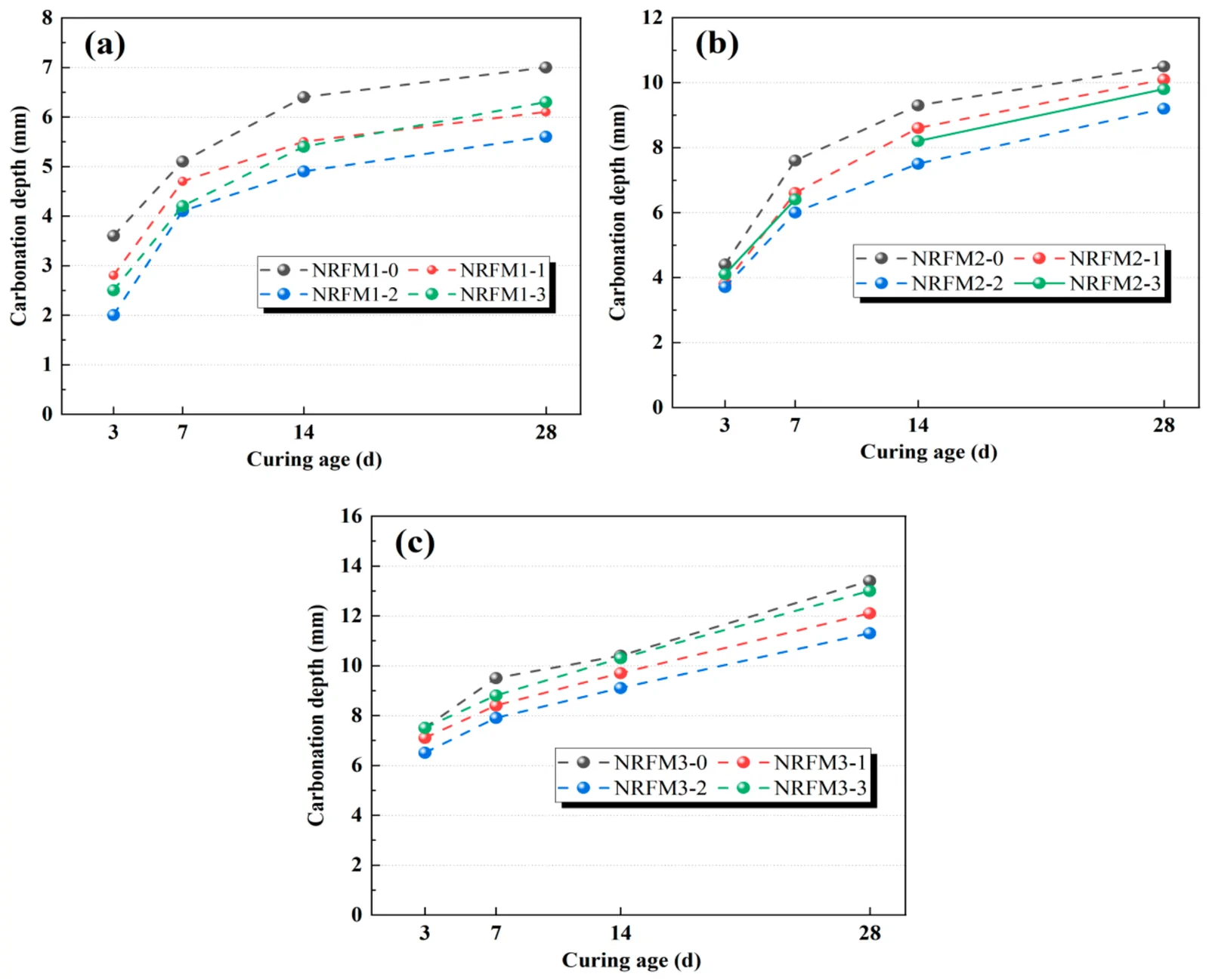
Carbonation depths of different series of NRFMs at different cement/sand ratios: (**a**) 1:1; (**b**) 1:2; (**c**) 1:3.

Compared with ordinary Portland cement, the hydration system of SAC contains low Ca(OH)_2_ content, and its hydration products are mainly C-A-H and AFt with a low Ca/Si ratio. Thus, carbonation directly occurs on AFt and C-A-H gels, leading to faster decomposition of hydration products and an accelerated development rate of early carbonation depth, which is further aggravated by the incorporation of RFAs. Figure 6 indicates that as the cement-to-sand ratio decreases, and the carbonation depth obviously increases. At 28 days, the carbonation depth of NRFM3-0 is much higher than that of NRFM1-0, which is directly related to the insufficient paste content, high porosity, and well-developed CO_2_ diffusion channels in the low cement-to-sand ratio group. As the NS concentration increases from 0% to 2%, the carbonation depth decreases continuously, while a slight increase is observed at the 3% NS suspension concentration. Compared with their respective control groups, the 28 d carbonation depths of NRFM1-2, NRFM2-2 and NRFM3-2 are reduced by approximately 20%, 13% and 15%, respectively. This improvement is attributed to the micro-filling effect of NS, which refines the capillary pore structure, as well as the additional hydration products formed by the pozzolanic reaction, which optimize matrix compactness and effectively retard CO_2_ penetration. Furthermore, excessive NS suspension concentration tends to agglomerate and introduce micro-defects, which in turn impairs carbonation resistance. This trend is highly consistent with the variations observed in mechanical performance and shrinkage properties of NRFM.

### 3.5. Frost-Resistance Performance of NRFM

**Figure 7:**
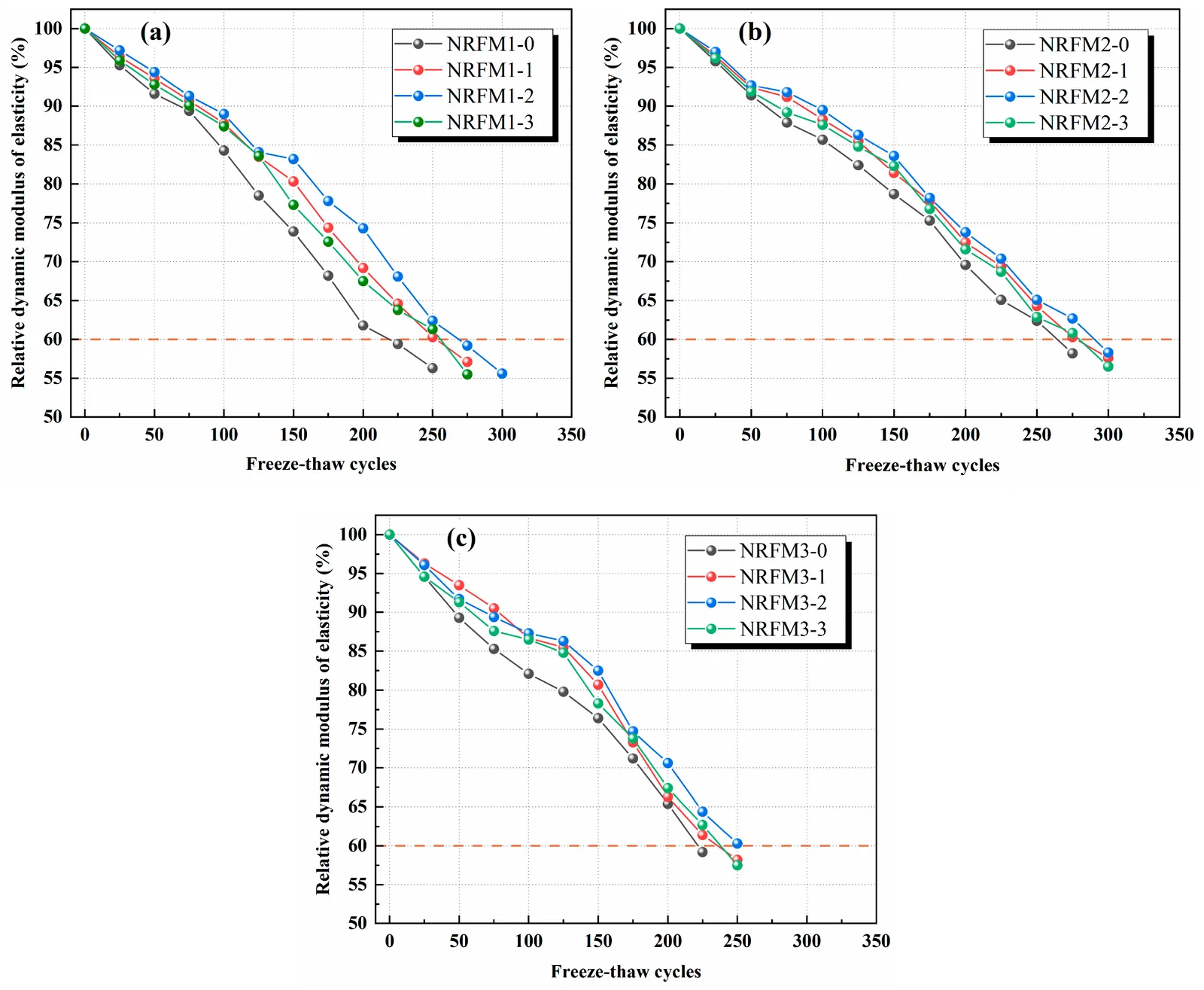
Relative dynamic modulus of elasticity of different series of NRFMs at different cement/sand ratios: (**a**) 1:1; (**b**) 1:2; (**c**) 1:3.

Figure 7 presents the evolution of the relative dynamic elastic modulus of nano-SiO_2_-modified recycled fine aggregate SAC mortar under freeze–thaw cycles. As shown in the figures, for mixtures with an identical sand–cement ratio, the incorporation of nano-SiO_2_ can significantly restrain the propagation of internal microcracks under freeze–thaw actions, improve the compactness of interfacial transition zones (ITZs), and markedly slow down the attenuation of the relative dynamic elastic modulus. The optimal nano-SiO_2_ dosage is determined to be 2%, while further increasing the dosage to 3% slightly weakens the modification effect due to the poor dispersion of excess nano-SiO_2_ particles. Meanwhile, at the same nano-SiO_2_ dosage, a higher content of recycled fine aggregates introduces more initial defects inside the mortar matrix, leading to deteriorated frost resistance. Nevertheless, the 2% optimal nano-SiO_2_ dosage can compensate for the freeze–thaw damage defects of mortars with high recycled-fine-aggregate replacement levels, as the interfacial filling effect and pozzolanic densification effect of nano-SiO_2_ can greatly narrow performance gaps.

### 3.6. Microstructural Properties of NRFM

#### 3.6.1. Thermogravimetric Analysis (TG/DTG)

**Figure 8:**
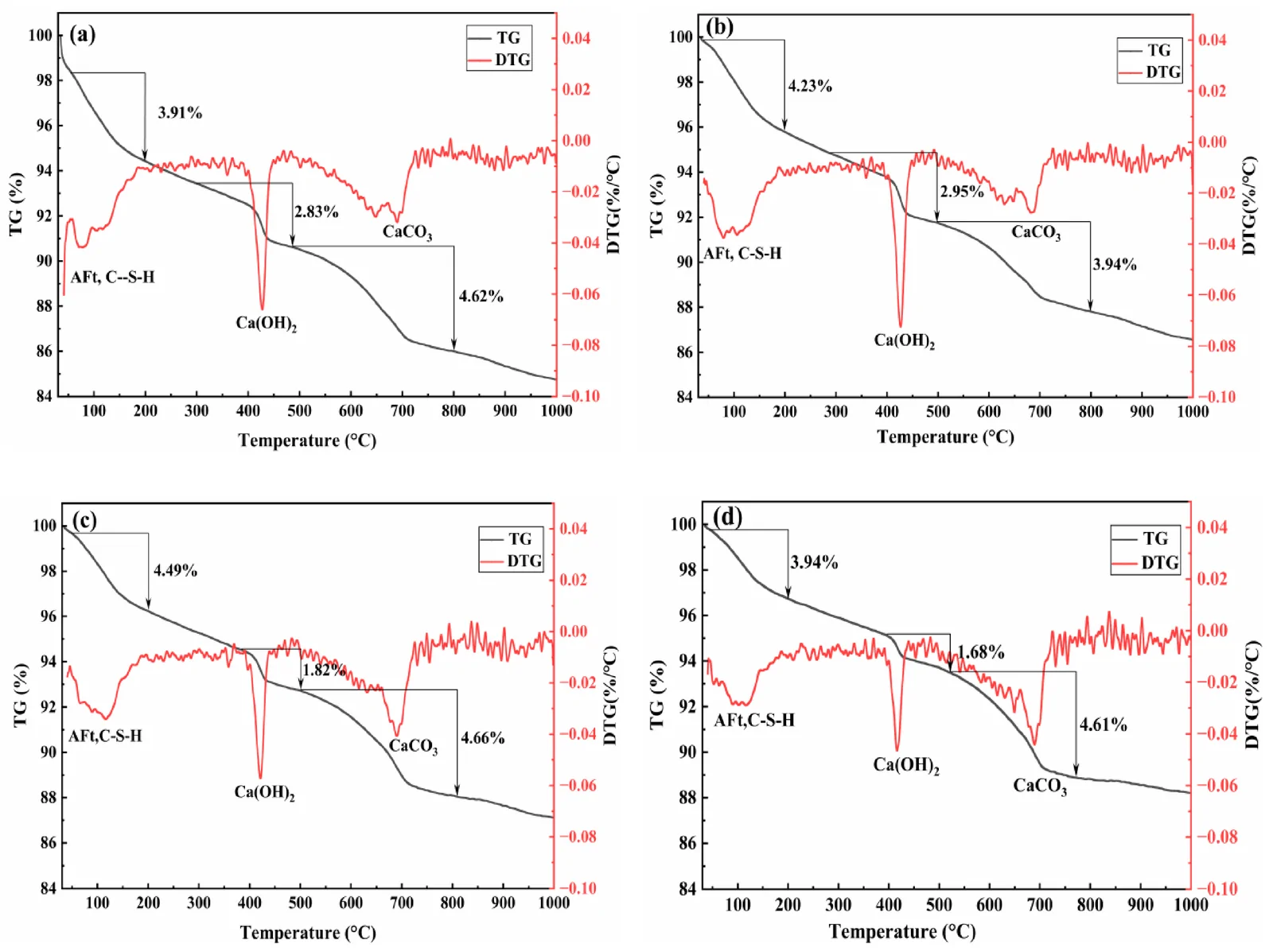
TG/DTG curves of NRFMs at different NS concentrations: (**a**) 0%; (**b**) 1%; (**c**) 2%; (**d**) 3%.

The NRFM mortar group with a cement sand ratio of 1:1 was selected as the micro testing group. Figure 8 presents the TG-DTG curves of NRFM at different NS concentrations with a cement–sand ratio of 1:1. Three distinct mass-loss stages are observed for all NRFM specimens at 50–300 °C, 400–600 °C and 600–800 °C, respectively.

The total mass loss ranging from 50 to 300 °C arises from the superposition of multiple overlapping dehydration reactions. This signal primarily covers the crystallization water of newly formed AFt, interlayer bound water of C-S-H gel generated by the NS pozzolanic reaction, as well as adsorbed pore water and low-temperature dehydration of aged C-S-H and minor AFm carried by residual old mortar on recycled fine aggregate surfaces. These multi-source dehydration processes overlap extensively and form a broad continuous DTG peak band in this temperature interval, as clearly observed in the DTG curves in Figure 8. Under the same RFA content and curing regimes across all groups, the interference from residual aggregate hydration products remains consistent among mixtures. Accordingly, the relative variation in total 50–300 °C mass loss with an increasing NS concentration still can reflect the changing yield of fresh SAC hydration products (AFt and C-S-H gel). As the NS concentration rises from 0% to 3%, the corresponding mass-loss ratios of the groups are 3.91%, 4.23%, 4.49% and 3.94%, showing an overall trend of first increasing and then decreasing. This confirms that 2% NS accelerates the hydration of SAC and maximizes the formation of AFt and C-S-H gel. At 3% NS, nanoparticle agglomeration deteriorates particle dispersion and weakens the hydration-accelerating effect, which leads to lower weight loss than the 2% NS group. However, all NS-modified samples exhibit higher mass loss than the blank control group, demonstrating that NS increases the total amount of hydration products in hardened paste.

The mass loss at 400–600 °C is attributed to the thermal decomposition of Ca(OH)_2_, and the corresponding mass-loss ratios of the four groups are 2.83%, 2.95%, 1.82% and 1.68%. For the 1–3% NS group, active silica participates in the pozzolanic reaction to consume Ca(OH)_2_ and form abundant C-S-H gel [43]. The blank sample lacks reactive SiO_2_ for Ca(OH)_2_ consumption and features fixed inherent Ca(OH)_2_ from recycled fine aggregates, hence its large weight loss. Moreover, the weight loss between 600 and 800 °C corresponds to decarbonation of CaCO_3_. With an increasing NS concentration, the variation of each group in CaCO_3_ weight loss is insignificant; despite nanoparticle agglomeration, the 3% NS specimen still possesses a denser matrix than the 1% NS and blank samples, thereby showing the lowest decarbonation weight loss.

#### 3.6.2. XRD Analysis

**Figure 9:**
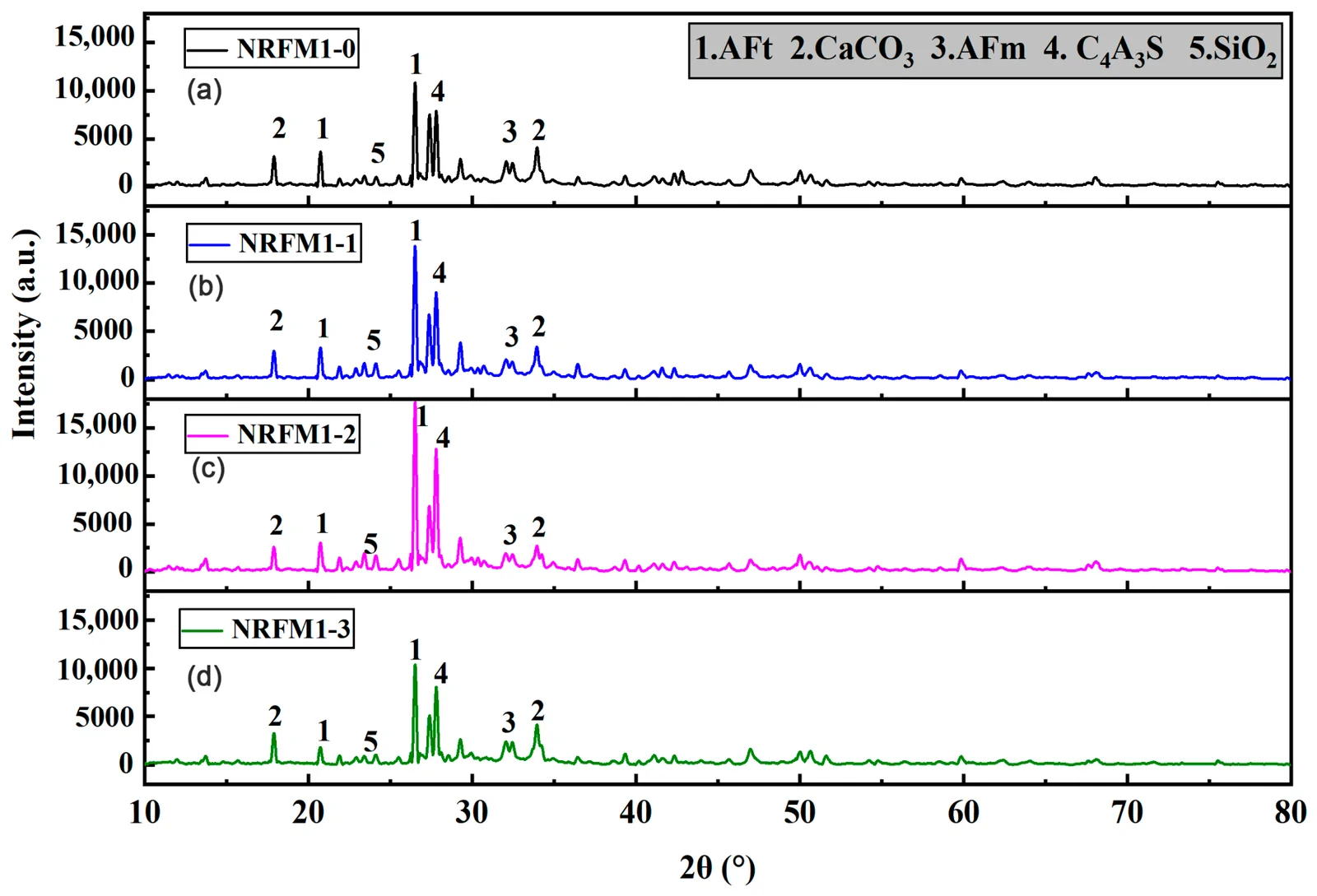
XRD patterns of NRFMs at different NS concentrations: (**a**) 0%; (**b**) 1%; (**c**) 2%; (**d**) 3%.

Figure 9 presents the XRD patterns of the NRFM1 series modified with different NS concentrations, which may reflect changes in the composition and relative content of hydration products. As shown in this figure, the crystalline phases mainly consist of calcite (CaCO_3_), ettringite (AFt), monosulfoaluminate (AFm), quartz (SiO_2_) and unhydrated calcium sulfoaluminate clinker (C_4_A_3_$\bar{S}$) [44]. C-S-H gel exists as amorphous gel without distinct sharp diffraction peaks. No emerging characteristic diffraction peaks are detected in all patterns, revealing that NS modification fails to produce new crystalline hydration products in NS-modified recycled fine aggregate mortar. As the NS concentration rises from 0% to 2%, the characteristic peaks of residual C_4_A_3_$\bar{S}$ gradually decline, while the AFt characteristic peak intensity increases continuously, with the maximum AFt peak value obtained for NRFM1-2 at the 2% NS concentration. This demonstrates that appropriate NS modification accelerates the hydration process of SAC paste and facilitates AFt formation by heterogeneous nucleation and pozzolanic reactivity. Nevertheless, excessive NS (3% concentration) leads to an obvious reduction in AFt characteristic peaks, implying inhibited hydration progress. The slight increase in the AFt characteristic peak at the 2% NS concentration is attributed to accelerated gypsum consumption, which triggers partial phase transformation from AFt to AFm. In addition, the CaCO_3_ peak intensity does not increase with growing NS concentration, and CaCO_3_ mainly originates from pre-carbonated components of recycled fine aggregates. However, as the cement-to-sand ratio decreases to 1:3, the RFA proportion increases, and the SAC paste decrease, which can easily lead to a decrease in the production of AFt. Overall, the XRD results are synergistic with the changes in macroscopic mechanics and durability performance, revealing the microscopic mechanism of NS modification in NRFM.

#### 3.6.3. Microhardness of NRFM

**Figure 10:**
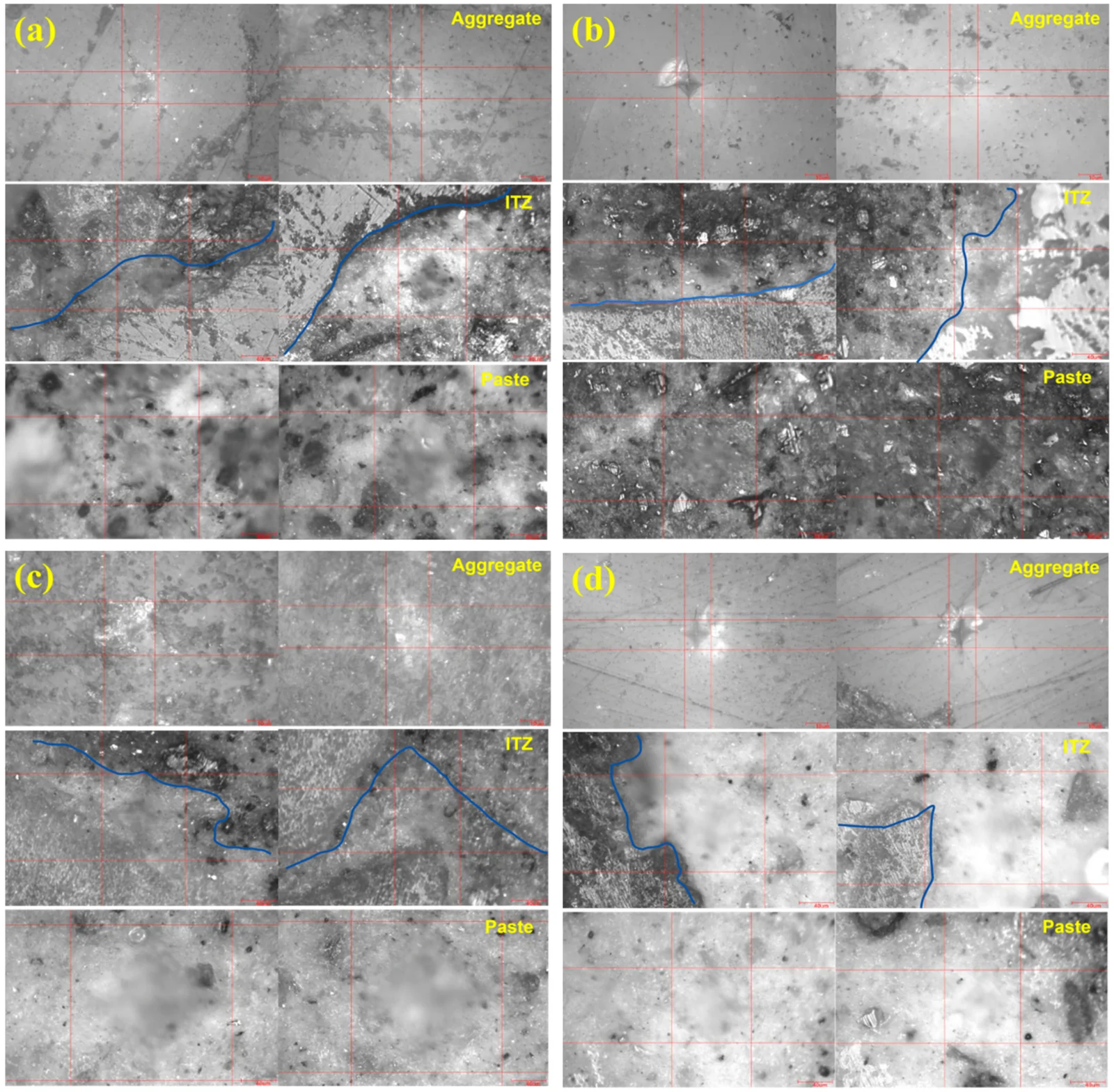
Micromorphology of NRFMs at different NS concentrations: (**a**) 0%; (**b**) 1%; (**c**) 2%; (**d**) 3%.

**Figure 11:**
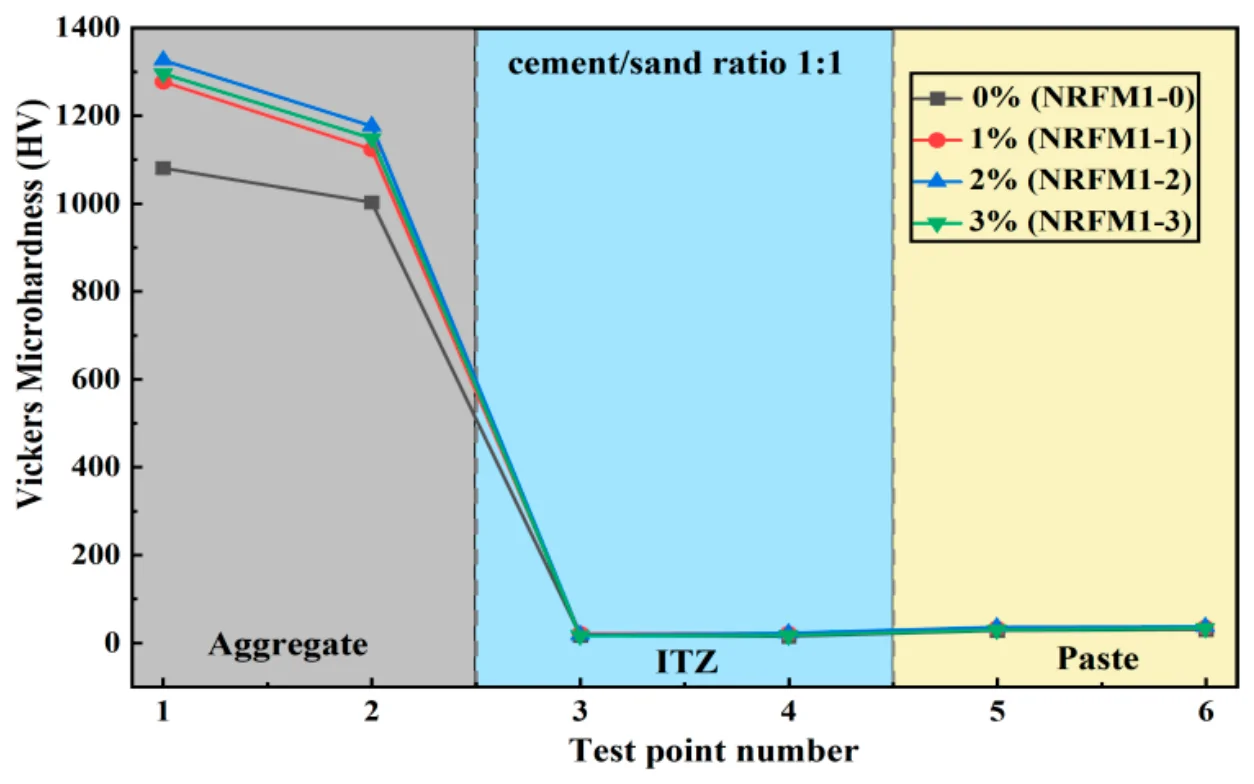
Microhardness values of NRFMs in various regions at different NS concentrations.

Figure 10 and Figure 11 present the micromorphology and microhardness of NS-modified SAC recycled mortars at a cement–sand ratio of 1:1. Six test points were selected for each specimen. Points 1–2 correspond to RFAs, points 3–4 denote the interfacial transition zone (ITZ), and points 5–6 refer to hardened cement paste. It can be seen that all specimens follow the hardness sequence: RFA > cement paste > ITZ. The ITZ acts as the mechanical weak zone of mortar, accompanied by a sharp drop in microhardness. NS modification effectively improves microhardness across all testing regions. As the NS concentration increases from 0% to 2%, the microhardness of the aggregate surface, ITZ and paste rises synchronously, with sample NRFM1-2 achieving the optimal hardness up to 1326 HV. Micromorphological observation confirms that the ITZ of the 2% NS-modified sample in Figure 10c possesses smooth and regular boundaries; pores and microcracks are fully filled with hydration products, resulting in a dense microstructure and maximum microhardness. However, further increasing the NS concentration to 3% causes NS agglomeration and poor dispersion, deteriorating the pore-filling effect and inducing a slight reduction in microhardness of the ITZ and paste, as the interface transition zone in Figure 10d shows a large white area of NS aggregation and flocculation, weakening the modification efficiency and causing lower microhardness of the NRFM paste compared with the 2% NS group.

#### 3.6.4. SEM Images of NRFM

**Figure 12:**
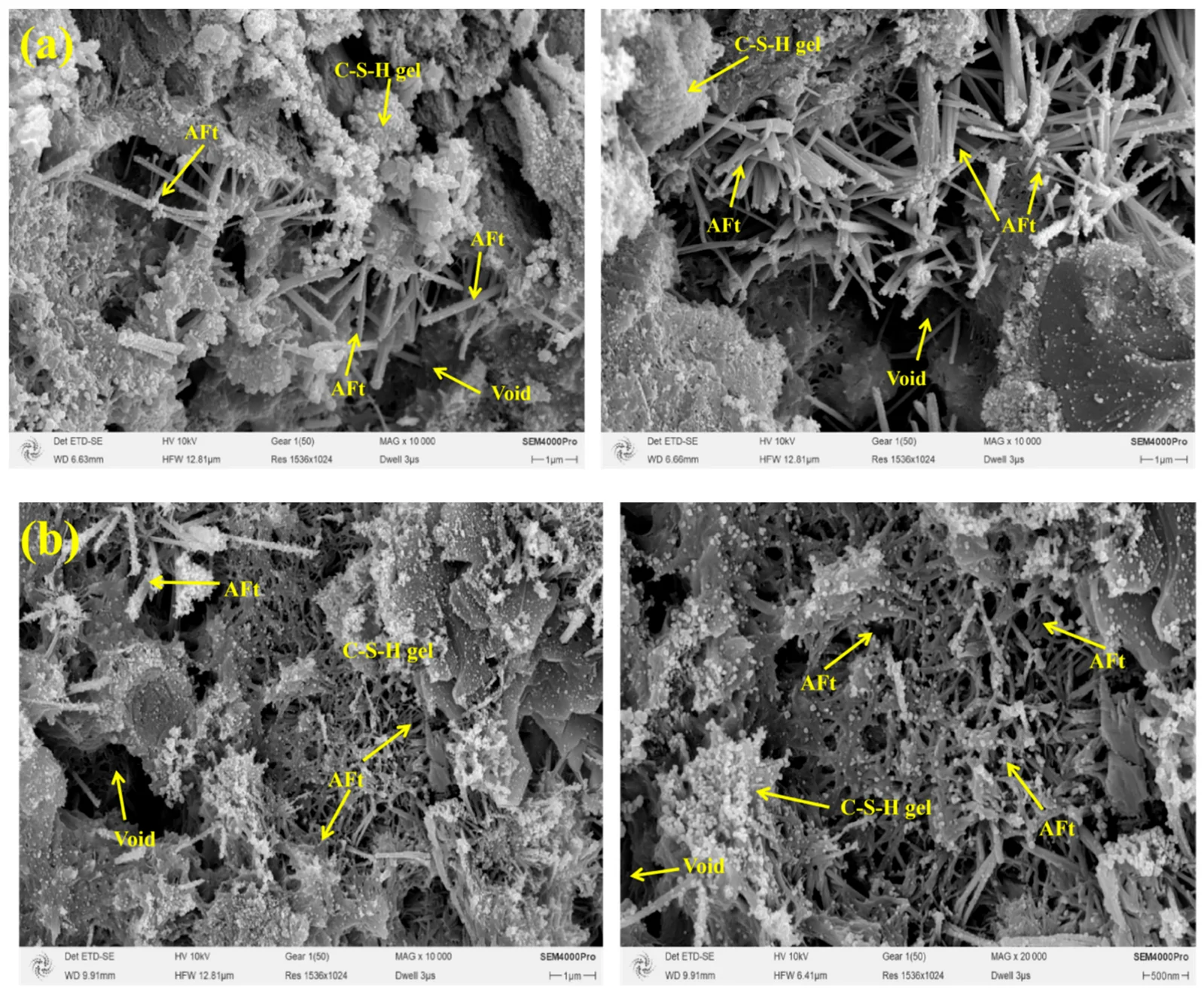

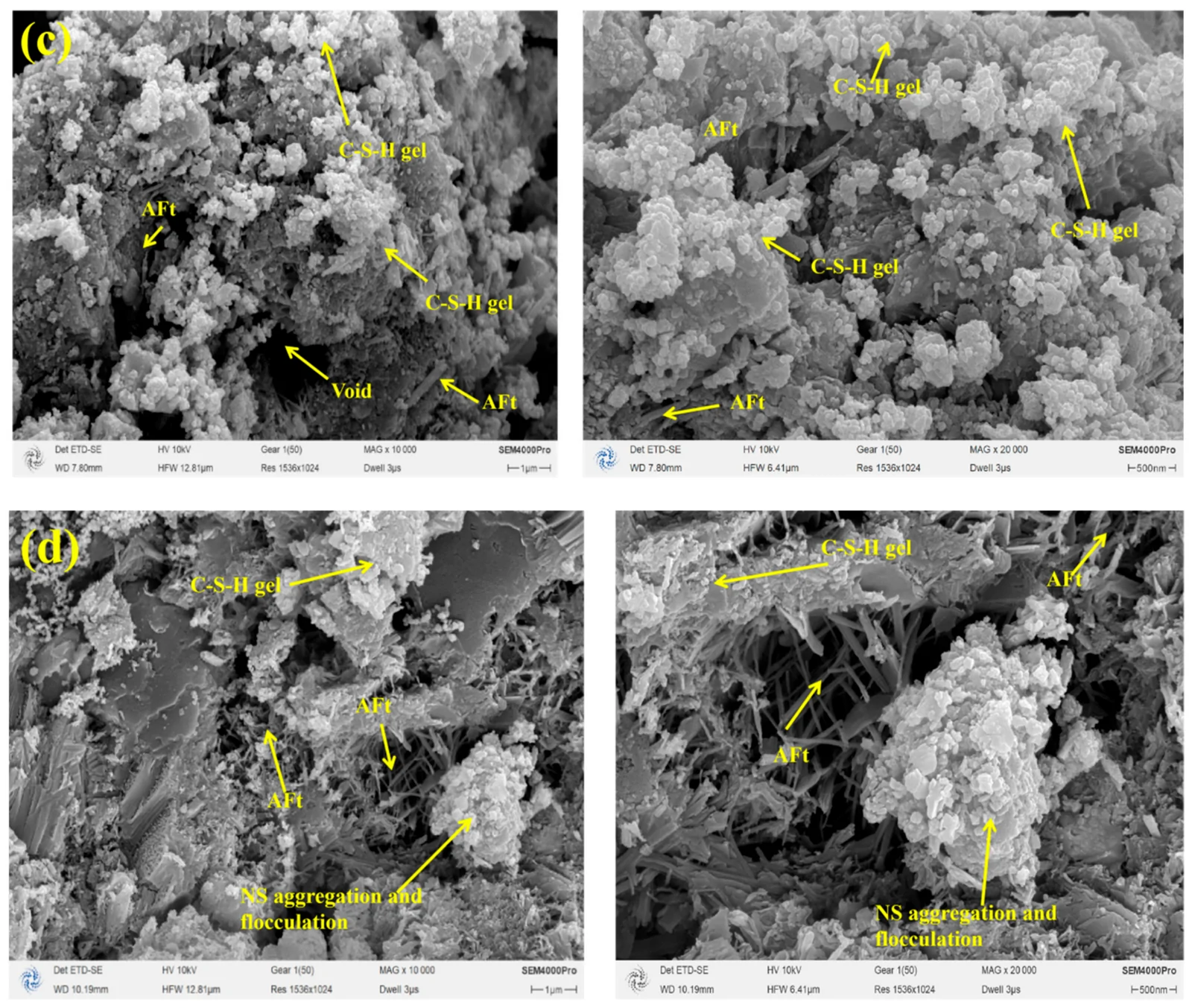
SEM morphology of NRFM at different NS concentrations: (**a**) 0%; (**b**) 1%; (**c**) 2%; (**d**) 3%.

As shown in Figure 12a, coarse rod-like AFt crystals are sparsely and irregularly distributed in the SAC control group sample, with loose interconnection among hydration products. Abundant pores and voids exist inside the paste, and the formed C-S-H gel is low in content and scattered in agglomerates, resulting in numerous pore defects within the paste. This is mainly attributed to insufficient SAC hydration, which causes inadequate gel filling in the ITZ and yields a relatively porous microstructure. From Figure 12b, it can be observed that compared with the control group, AFt grains are further refined and increased in number at 1% NS modification, the acicular crystals arrange more compactly, and the amount of flocculent C-S-H gel wrapped around AFt rises to fill part of the large pores. Both the size and quantity of pores in the paste decrease remarkably, while a small amount of interconnected micropores still remains. As illustrated in Figure 12c, when the NS concentration rises to 2%, acicular AFt crystals are tightly encapsulated by abundant flocculent C-S-H gel, and fine NS particles disperse uniformly throughout the matrix. Hardly any obvious macropores can be found inside the SAC paste, and cross-linked hydration products construct a continuous and dense skeleton with sufficient pore filling in the ITZ. The results reveal that the combined nucleation effect and pozzolanic reaction of NS accelerate SAC hydration at the 2% NS concentration, maximizing the production of AFt and C-S-H, which coincides with the analytical results of the XRD, TG/DTG and microhardness tests. However, Figure 12d reveals that excessive NS concentration triggers particle agglomeration accompanied by obvious aggregated nano-SiO_2_ clusters in local regions. The uneven distribution of hydration products regenerates large pores inside the paste, leading to poorer paste compactness compared with the specimen at the 2 NS concentration.

#### 3.6.5. SEM-EDS Analysis of NRFM

**Figure 13:**
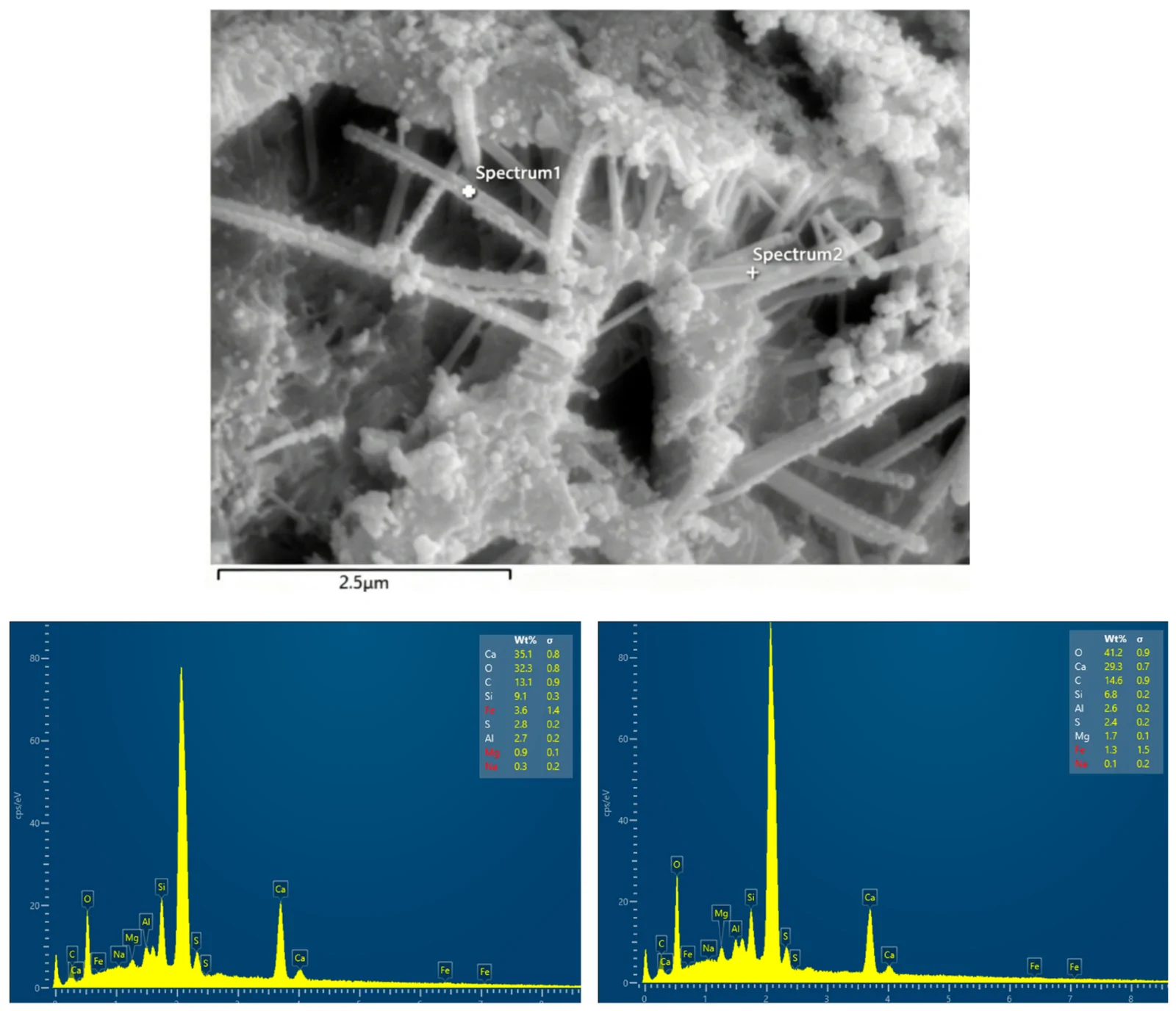
SEM-EDS analysis of NRFM at 0% NS concentration.

**Figure 14:**
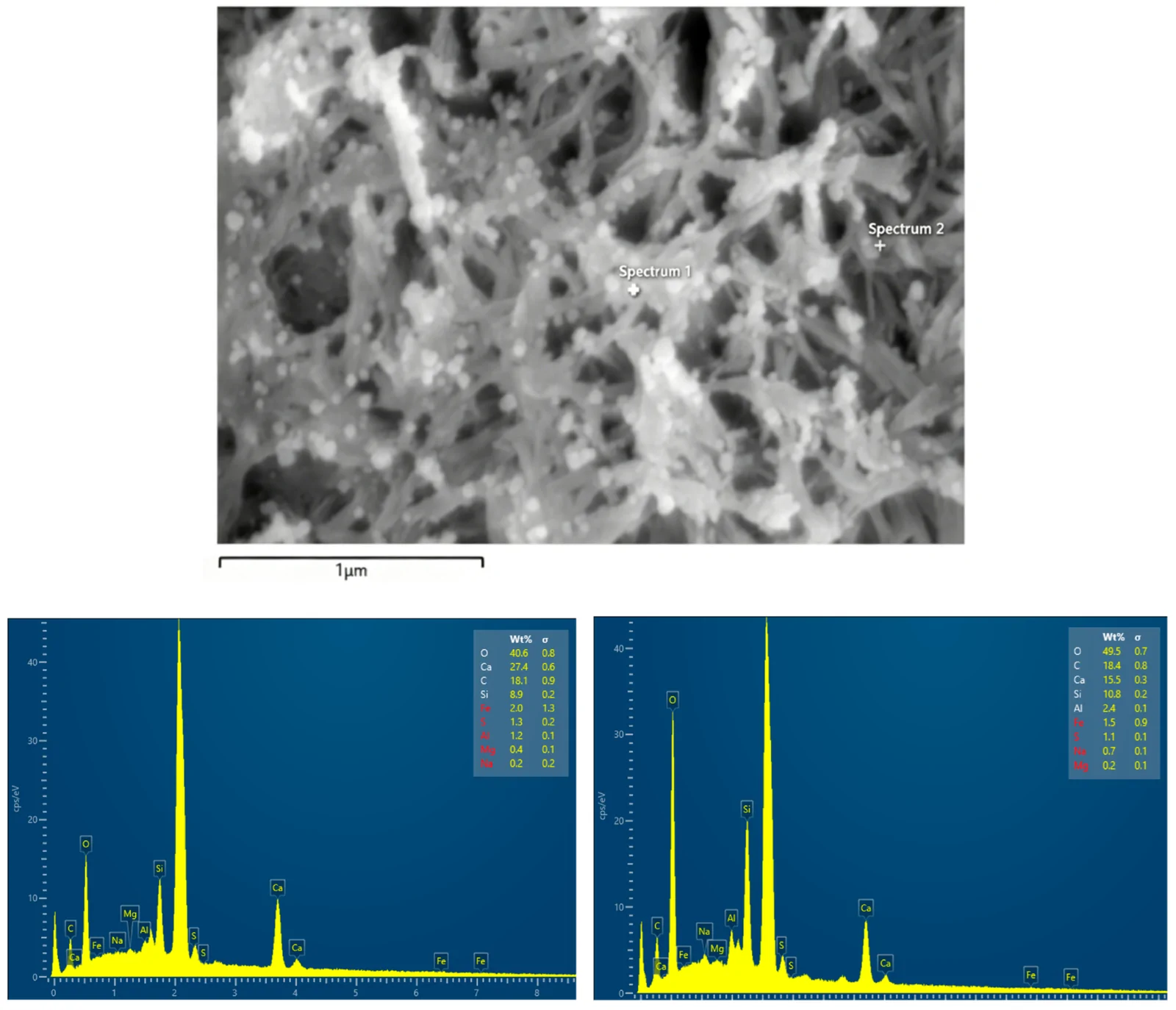
SEM-EDS analysis of NRFM at 1% NS concentration.

**Figure 15:**
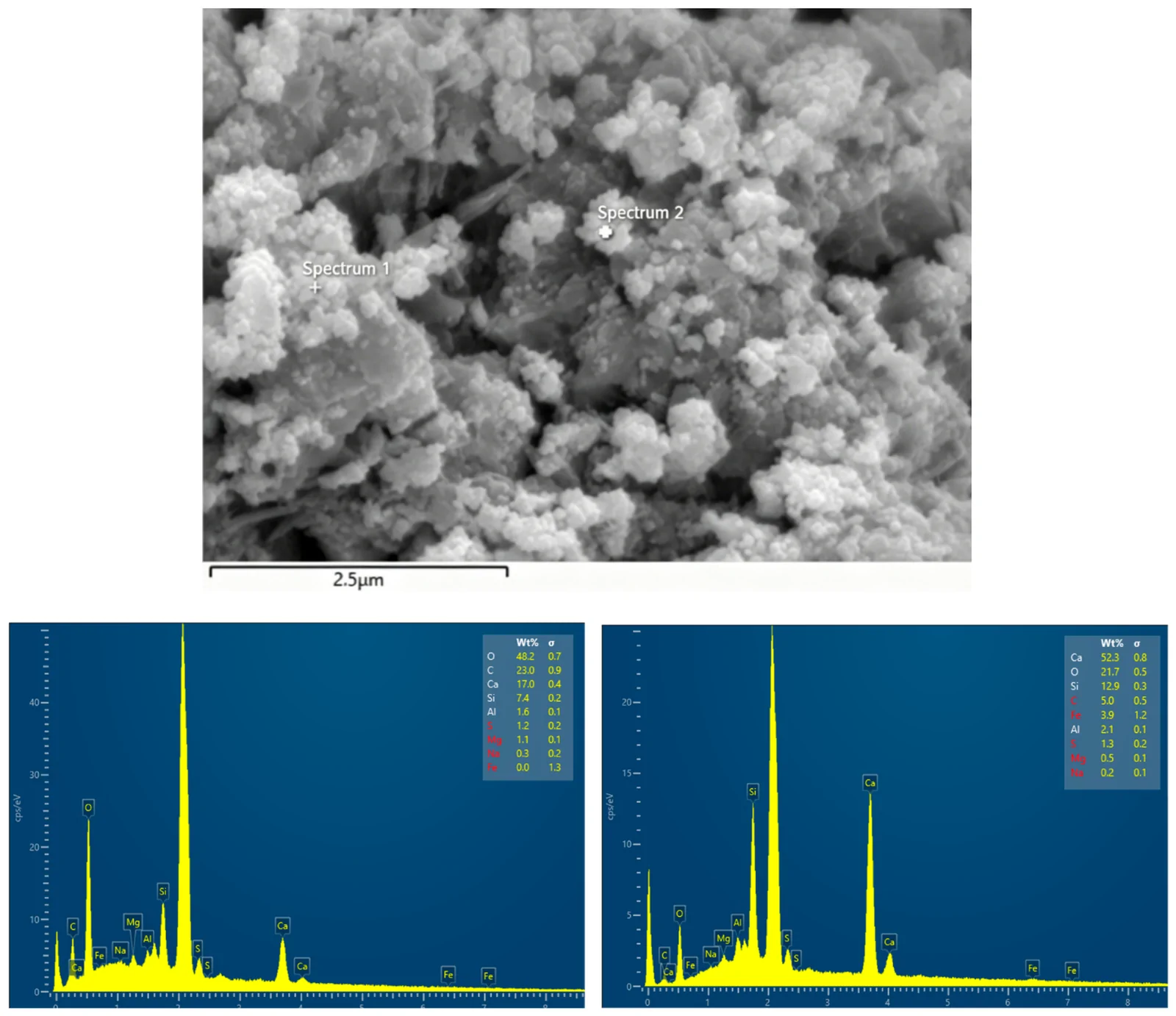
SEM-EDS analysis of NRFM at 2% NS concentration.

**Figure 16:**
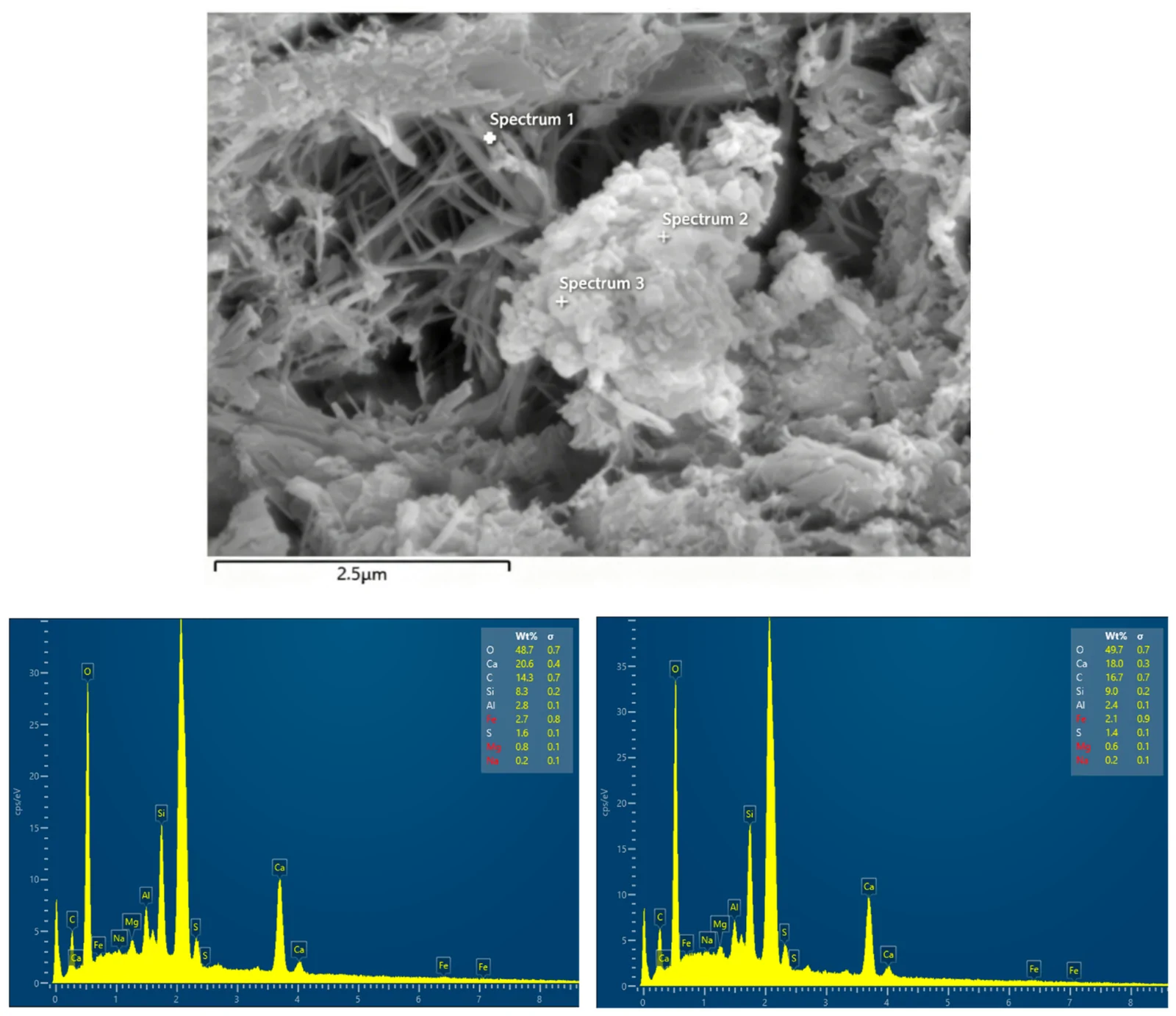
SEM-EDS analysis of NRFM at 3% NS concentration.

Combining SEM morphology and semi-quantitative EDS elemental results, the elemental evolution of hydration products for NRFM modified with 0–3% NS is presented in Figure 13, Figure 14, Figure 15 and Figure 16. For the control specimen in Figure 13, Spectrum 1 was positioned on AFt crystals and Spectrum 2 on the flocculent gel phase. Spectrum 1 is dominated by characteristic elements of Ca, S, Al and O, verifying the tested phase is AFt [45]. The amorphous agglomerates surrounding AFt consist of aluminum hydroxide gel AH_3_ and C-S-H gel with limited Si and Al concentrations, and no C-A-S-H gel is formed due to insufficient reactive silica to drive the pozzolanic reaction, resulting in a low gel amount and abundant voids within the hardened paste. As shown in Figure 14, the Si content rises simultaneously at both detection points, with a more prominent increment observed in the gel region. Dissolved reactive silica from NS participates in the pozzolanic reaction, refining the AFt grain size and improving the compactness. It can be seen from Figure 15 that the specimen modified by 2% NS achieves the optimal formulation. Acicular AFt crystals are fully encapsulated by abundant flocculent gel with barely exposed ettringite, while fine NS nanoparticles disperse homogeneously throughout the gel matrix. The Si content at the gel spot peaks at 12.9% across all tested groups, accompanied by an obvious decline in the Ca/Si ratio. Consumptions of Ca(OH)_2_ by reactive NS convert C-S-H gel into the predominant gel phase, and the elevated Si concentration at the AFt site also indicates uniform coating of AFt by C-S-H, with micro-pores sufficiently filled by hydration products. However, as shown in Figure 16, 3% NS triggers severe nanoparticle agglomeration and accumulation of unreacted NS, leading to highly divergent Si contents at different gel locations with abnormally high silicon in partial domains. Such agglomeration reduces the utilization efficiency of effective reactive silica.

#### 3.6.6. Microscopic Mechanism Analysis of NRFM

**Figure 17:**
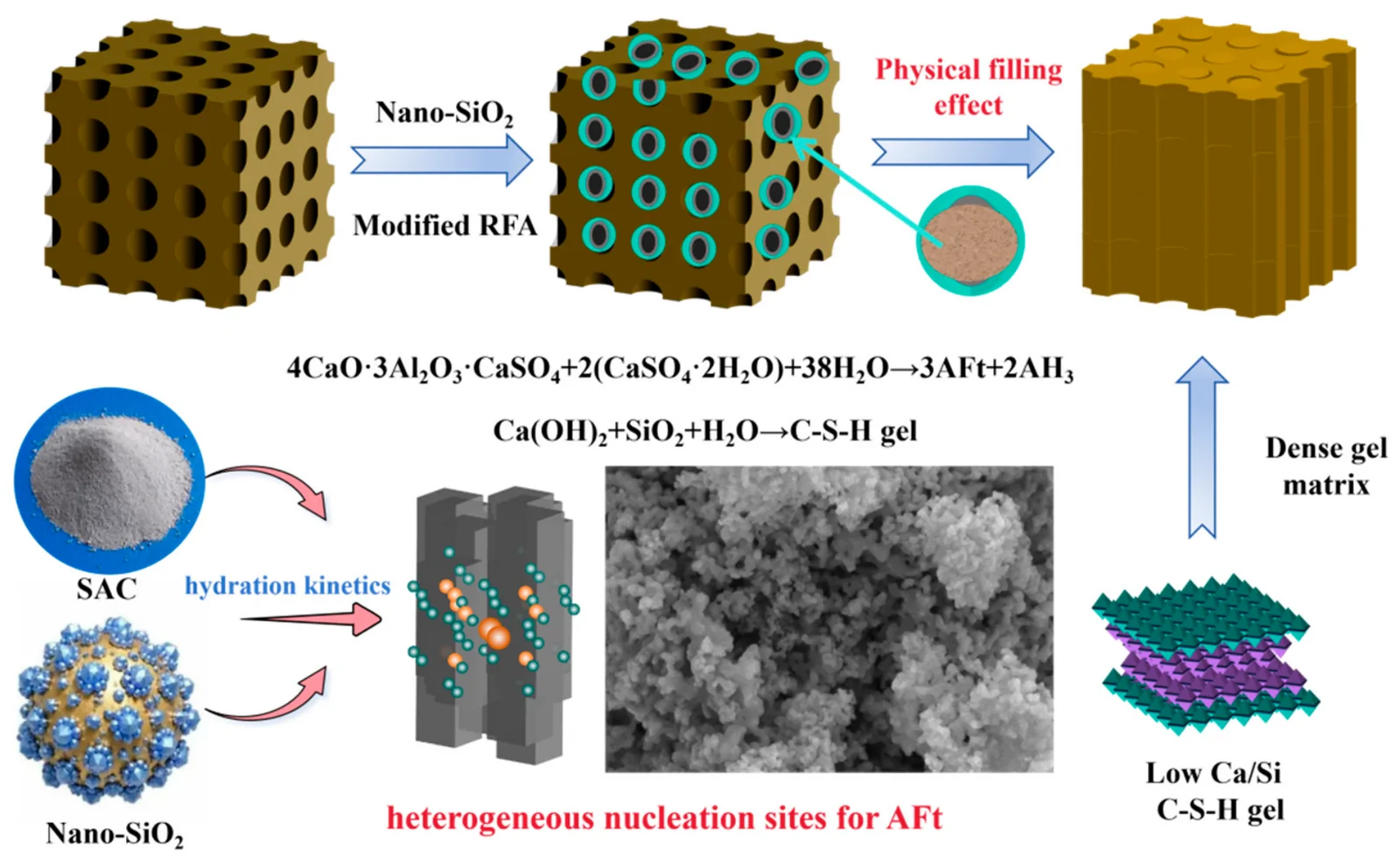
Microscopic mechanism analysis of NS-modified NRFM.

The microscopic mechanism is illustrated in Figure 17. On the one hand, nano-SiO_2_ particles fill micro-pores and microcracks on the surface of RFAs from the physical filling effect. Meanwhile, the pozzolanic reaction between NS and portlandite accumulated at the ITZ consumes oriented Ca(OH)_2_ crystals and generates low-density Ca/Si C-S-H gel. The optimal NS suspension concentration is 2%, while the 3% content leads to particle agglomeration and introduces secondary pores, thus weakening the modification efficiency. On the other hand, NS serves as heterogeneous nucleation sites to accelerate the hydration kinetics of SAC [46]. It regulates the equilibrium of liquid ions to restrain the overgrowth of AFt, and the supplementary active silicon source promotes the continuous formation of C-S-H gel in the long term. The refined pore structure inhibits the propagation of internal microcracks, which can enhance the performance of modified samples.

## 4. Conclusions

In this study, recycled fine aggregates whose surface was modified with different nano-SiO_2_ concentrations were prepared, and rapid hardening sulfoaluminate cement (SAC) was used as the cementitious material. The mechanical properties, durability, and micro-structure evolution of SAC-based nano-modified recycled fine aggregate mortar were systematically studied. The obtained conclusions are as follows:

(1) Nano-SiO_2_ (NS) pre-soaking surface modification effectively improves the macroscopic performance of SAC recycled fine aggregate mortar. As the NS concentration increases from 0% to 2%, mechanical strength and carbonation resistance are gradually enhanced, while shrinkage is reduced. A 2% NS concentration is the optimal concentration, and 3% NS causes nanoparticle agglomeration to degrade modification efficiency. Moreover, NS presents a more remarkable effect on mortars with low cement–sand ratios.
(2) Appropriate NS concentration promotes an SAC hydration reaction by the nucleation effect and pozzolanic reaction, increases the generation of AFt and C-S-H gel and consumes Ca(OH)_2_, and the weight loss rate of Ca(OH)_2_ reduced from 2.95% to 1.68%, while excessive 3% NS agglomeration restrains hydration and leaves more initial defects inside the matrix.
(3) Microhardness and SEM results confirm the universal hardness order: recycled aggregate > cement paste > ITZ, and ITZ is the intrinsic weak region of SAC recycled mortar. A 2% NS modification fills ITZ pores with abundant hydration products, smoothens the aggregate–paste boundary and maximizes interfacial microhardness up to 1326 HV, whereas 3% NS agglomerates form new internal defects and deteriorate interfacial compactness.
(4) SEM-EDS elemental analysis verifies that a rising NS concentration increases the silicon content in hydration products and reduces the Ca/Si ratio, as the Si content at the gel spot at a 2% NS concentration reaches 12.9%, and acicular AFt crystals are fully encapsulated by abundant flocculent gel with barely exposed ettringite. NS surface modification can realize high-value resource utilization of waste recycled fine aggregates in a rapid-hardening sulfoaluminate cement system.

## Figures and Tables

**Table 1:** The detailed physical performance indexes of SAC.

Cement Type	Specific Surface Area/m^2^·kg^−1^	Initial Setting Time/min	Final Setting Time/min	Density /kg·m^−3^	Flexural Strength/MPa		Compressive Strength/MPa	
					3 d	28 d	3 d	28 d
SAC	453	26	45	3174	6.4	7.9	35.2	44.7

**Table 2:** The XRF chemical composition of SAC (%).

Cement	CaO	SiO_2_	Al_2_O_3_	Fe_2_O_3_	MgO	SO_3_	Na_2_O	TiO_2_	Loss on Ignition (LOI)
SAC	55.37	9.56	18.44	3.32	4.15	5.69	0.71	0.60	1.67

**Table 3:** Physical properties of raw and NS-modified recycled fine aggregates.

NS Suspension Concentration	24 h Water Absorption/%	Apparent Density/(kg/m^3^)	Bulk Density/(kg/m^3^)	Crushing Index/%
Unmodified RFA	7.05	2538	1457	16.14
1% NS-modified RFA	6.24	2567	1492	14.98
2% NS-modified RFA	5.53	2588	1523	13.35
3% NS-modified RFA	5.86	2571	1504	14.22

**Table 4:** Mix proportion designs of NRFM mortar.

Testing Number	Cement–RFA Ratio	Mass Concentration of NS Suspension (%)	Cement (kg/m^3^)	Recycled Fine Aggregate (kg/m^3^)	Actual Water Content (kg/m^3^)	Water Reducer (kg/m^3^)
NRFM1-0	1:1	0	900	900	450	9.0
NRFM1-1		1	900	900	456	9.0
NRFM1-2		2	900	900	462	9.0
NRFM1-3		3	900	900	468	9.0
NRFM2-0	1:2	0	600	1200	302	6.0
NRFM2-1		1	600	1200	307	6.0
NRFM2-2		2	600	1200	313	6.0
NRFM2-3		3	600	1200	319	6.0
NRFM3-0	1:3	0	450	1350	227	4.5
NRFM3-1		1	450	1350	231	4.5
NRFM3-2		2	450	1350	237	4.5
NRFM3-3		3	450	1350	241	4.5

## Data Availability

The original contributions presented in this study are included in the article. Further inquiries can be directed to the corresponding authors.
